# Oxidative Stress Footprints in Bone Marrow Mesenchymal Stem Cells from Untreated Advanced Breast Cancer

**DOI:** 10.32604/or.2026.074321

**Published:** 2026-03-23

**Authors:** Francisco Raúl Borzone, María Belén Giorello, Agustina Freire, Leandro Marcelo Martinez, Leonardo Feldman, Federico Dimase, Pablo Evelson, Irene Larripa, Emilio Batagelj, Marcela Beatriz González Cid, Norma Alejandra Chasseing

**Affiliations:** 1 Laboratorio de Inmunohematología, Instituto de Biología y Medicina Experimental (IBYME), Fundación IBYME, Consejo Nacional de Investigaciones Científicas y Técnicas (CONICET), Ciudad Autónoma de Buenos Aires, Argentina; 2 Laboratorio de Medicina Regenerativa Cardiovascular, Instituto de Medicina Traslacional, Trasplante y Bioingeniería (IMETTyB-Universidad Favaloro-CONICET), Ciudad Autónoma de Buenos Aires, Argentina; 3 Instituto de Bioquímica y Medicina Molecular (IBIMOL), Facultad de Farmacia y Bioquímica, CONICET, Universidad de Buenos Aires, Ciudad Autónoma de Buenos Aires, Argentina; 4 Division of Hematology and Medical Oncology, Department of Medicine, Weill Cornell Medical College, New York, NY, USA; 5 Facultad de Ciencias de la Salud, Universidad Nacional del Centro de la Provincia de Buenos Aires (UNCPB), Tandil, Provincia de Buenos Aires, Argentina; 6 Servicio de Hematología, Hospital Militar Central, Ciudad Autónoma de Buenos Aires, Argentina; 7 Laboratorio de Genética Hematológica, Instituto de Medicina Experimental (IMEX)-CONICET, Academia Nacional de Medicina, Ciudad Autónoma de Buenos Aires, Argentina; 8 Servicio de Oncología, Hospital Militar Central, Ciudad Autónoma de Buenos Aires, Argentina; 9 Laboratorio de Mutagénesis, IMEX-CONICET, Academia Nacional de Medicina, Ciudad Autónoma de Buenos Aires, Argentina

**Keywords:** Mesenchymal stem/stromal cells, bone marrow, breast cancer, reactive oxygen species damage, secretome, pre-metastatic niche, bone metastasis

## Abstract

**Backgrounds:**

Breast cancer metastasis remains the leading cause of mortality and frequently targets the bone. Breast cancer cells release soluble factors and extracellular vesicles that disrupt bone marrow (BM)/bone homeostasis, promoting osteoclastogenesis and the accumulation of senescent cells. In line with updated cancer hallmarks, senescent mesenchymal stem/ stromal cells (MSCs), osteoblasts, and osteocytes contribute to remodeling of the BM microenvironment, thereby favoring pre-metastatic niche (PMN) formation and subsequent bone metastasis. We previously demonstrated that untreated stage III-B breast cancer patients (BCPs) exhibit increased oxidative stress and elevated reactive oxygen species (ROS) levels, accompanied by senescent and functionally impaired BM-MSCs—key regulators of BM/bone homeostasis. In the present study, we sought to identify the molecular targets affected by oxidative stress that drive MSC senescence in these patients.

**Methods:**

BM-MSCs were isolated from untreated stage III-B BCPs and healthy volunteers (HVs). Oxidative stress responses were evaluated by quantitative real-time PCR (qRT-PCR) analysis of stress- and antioxidant-related genes. Oxidative damage to DNA, proteins, and lipids was assessed using alkaline comet assay, chromosomal aberration (CAs) analysis, micronuclei (MN) and nuclear blebs (NBs) quantification, protein carbonyl content, and detection of 4-hydroxynonenal (4-HNE) adducts. The MSC secretome was analyzed by label-free quantitative proteomics followed by Gene Ontology enrichment analysis.

**Results:**

Our results show that elevated oxidative stress in BCPs induces the overexpression of oxidative stress–related and antioxidant response genes in BM-MSCs; however, this response is insufficient to prevent extensive ROS-induced damage to deoxyribonucleic acid (DNA), proteins, and lipids. In addition, proteomic analysis of the BM-MSC secretome revealed a distinct protein expression profile in BCPs compared with HVs.

**Conclusions:**

Together, these findings highlight oxidative stress–induced MSC damage as a key mechanism contributing to PMN formation and suggest potential therapeutic targets to mitigate bone metastasis in advanced breast cancer.

## 1 Introduction

Distant metastasis remains the leading cause of mortality among BCPs. Breast cancer metastases display marked organotropism, with bone representing the most frequent initial site, followed by lung and, to a lesser extent, liver and brain [1,2]. Importantly, bone metastases not only drive skeletal-related complications but also facilitate secondary dissemination of tumor cells through horizontal cross-metastatic seeding [3,4].

Primary breast tumors secrete soluble factors and extracellular vesicles that play a critical role in preparing and conditioning distant tissues for PMN formation [5–8]. In the BM/bone microenvironment, this paracrine signaling alters the behavior of osteoblasts and osteoclasts, promoting changes that support tumor cell colonization [9–11]. In parallel, it modulates MSC phenotype and function, inducing alterations that further contribute to the establishment of a permissive PMN [12–14].

Recent evidence highlights cellular senescence as a key driver of bone metastatic disease. In established breast cancer bone metastases, osteocytes undergo premature senescence and acquire a distinct senescence-associated secretory phenotype (SASP) that promotes bone degradation. These findings identify cellular senescence as an initiating event in lytic bone disease associated with metastatic breast cancer [15].

MSCs are central regulators of BM/bone homeostasis and key components of the PMN. In previous work, we demonstrated that MSCs isolated from the BM of untreated advanced BCPs (stage III-B, without detectable BM/bone metastasis) exhibit pronounced morphological alterations, including enlarged and flattened morphology driven by cytoskeletal rearrangements—early hallmarks of cellular senescence [16–18]. These cells also display increased senescence-associated β-galactosidase activity, reduced self-renewal and proliferative capacity, and impaired cell cycle progression, consistent with a senescent phenotype [16,19].

In addition to these functional changes, MSCs from advanced BCPs exhibit elevated oxidative stress, characterized by increased total and mitochondrial ROS production, reduced expression of human telomerase reverse transcriptase (TERT), and impaired telomere maintenance. This phenotype is accompanied by marked transcriptional alterations, including decreased expression of stemness-associated genes (OCT4, SOX2, and TERT), reduced fibroblastic colony-forming unit (CFU-F) capacity, impaired osteogenic and adipogenic differentiation, and enhanced pro-inflammatory and pro-osteoclastogenic activity [16,20–22].

Consistent with these findings, we previously detected increased expression of genes associated with osteoclastogenesis, inflammation, and tumor promotion, including C-C motif chemokine 2 (CCL2), macrophage colony-stimulating factor, and interleukin-6 (IL-6) [16]. Notably, IL-6 and CCL2 are prominent components of the SASP and are abundantly secreted by senescent cells [23]. In parallel, alterations in soluble factor levels were observed in both peripheral blood and BM plasma from BCPs, implicating these factors in breast cancer cell intravasation, extravasation, migration, proliferation, and bone resorption [21]. Moreover, conditioned media derived from MSC-CFU-F cultures of untreated advanced BCPs enhanced the migratory capacity of human breast cancer cell lines, supporting a direct role for MSCs in facilitating tumor cell homing to the BM/bone microenvironment [21].

Among the various stressors capable of inducing senescence in MSCs, oxidative stress represents a major driver. Oxidative stress arises from an imbalance between ROS production and antioxidant defenses [24,25]. We previously reported that untreated advanced BCPs exhibit a heightened oxidative state, as assessed by elevated thiobarbituric acid–reactive substances (TBARS) and increased ROS levels in both BM plasma and peripheral blood plasma [26]. Excessive ROS generation—largely originating from mitochondrial dysfunction—damages DNA, proteins, and lipids, thereby activating oxidative stress response pathways, including heat shock protein 90 alpha (HSP90) and nuclear factor erythroid 2-related factor 2 (Nrf2), as well as downstream antioxidant genes such as superoxide dismutase 1 (SOD1), superoxide dismutase 2 (SOD2), catalase (CAT), and glutathione peroxidase 1 (GPx1). When these protective responses are insufficient, sustained molecular damage promotes MSC dysfunction and senescence [27,28].

The BM is composed of multiple interconnected compartments that must remain functionally integrated to preserve overall BM/bone homeostasis. MSCs are essential to this process, as they regulate hematopoiesis and bone metabolism, including both bone formation and resorption. Accordingly, the maintenance of a sufficient and functional MSC pool is critical to prevent PMN establishment and subsequent colonization by breast cancer cells [12].

Although oxidative stress-induced damage in the BM microenvironment of BCPs is gaining increasing attention, its impact on MSCs remains poorly understood. This study aims to characterize the metabolic and molecular alterations in BM-derived MSCs from untreated advanced BCPs, including damage to DNA, proteins, and lipids, as well as the expression of genes involved in the oxidative stress response. Furthermore, it seeks to investigate how these alterations are reflected in the MSC secretome and their potential contribution to a pro-tumoral microenvironment in this patient group. Thus, we hypothesized that oxidative stress promotes molecular damage in BM-derived MSCs, facilitating PMN formation in breast cancer.

Targeting oxidative stress and its consequences on MSCs could offer new therapeutic avenues to counteract pro-tumoral microenvironment development.

## 2 Material and Methods

### 2.1 Patients

We conducted a prospective study including 10 patients diagnosed with breast cancer confirmed by histology and 10 HVs as controls. Inclusion criteria was women aged 50 to 65 years with invasive ductal breast carcinoma, clinical-pathological stage III-B, non-inflammatory (according to the TNM staging system of the International Union Against Cancer), with no prior surgery for the primary tumor, free of oncological treatment (chemotherapy, hormone therapy, immunotherapy, and/or radiotherapy), and in a menopausal state without hormonal treatment. The exclusion criteria included metastasis to the bone and/or BM or other organs, the presence of another primary tumor of the same or different origin, and diseases that could alter bone metabolism, such as osteoporosis, vitamin D deficiency, thyroid disease, parathyroid disease, and kidney damage. The absence of bone metastasis was confirmed by X-ray and bone scintigraphy, following the recommendations of the clinical guidelines established by the European Society for Medical Oncology (ESMO) [29]. Patients were selected without considering the molecular subtype of the breast tumor, given that the study group is very limited according to all the previously described inclusion criteria.

The control cohort consisted of women 45–65 years old, donors for allogeneic BM transplantation or HVs who attended the Hematology Service and had no involvement of the BM, immuno-hematological disorders, or bone issues, meaning they had no diseases that could affect bone metabolism. HVs were matched for menopausal status with breast cancer patients.

This study was conducted in accordance with the ethical principles of the Declaration of Helsinki. Ethical approval was obtained from the relevant institutional ethics committees of all participating institutions. Specifically, the study was approved by the *Comité de Ética Dr. Enrique T. Segura*, IBYME-CONICET (Project CE 051/2015), and by the *Comité Institucional de Revisión de Ensayos Clínicos (CIREC)*, Hospital Militar Central (Acta 326/2014). Written informed consent was obtained from all individual participants included in the study.

### 2.2 Isolation and Preparation of BM-MSCs

BM aspirates were obtained from the posterior iliac crest under local anesthesia and collected in preservative-free heparinized saline (25 units/mL; cat. 15077-019, Gibco, Grand Island, NY, USA). Mononuclear cells were isolated from BM samples using a Histopaque (density = 1.075 g/cm^3^; cat. 10771, Sigma Aldrich, St. Louis, MO, USA) density gradient. After centrifugation for 25 min at 340× *g*, the light-density BM mononuclear cell (MNC) fraction was recovered from the interface, washed twice with phosphate-buffered saline (PBS; 137 mM NaCl [cat. 2000164600, Biopack Productos Químicos, Zárate, BA, Arg]; 2.7 mM KCl [cat. 2000934600, Biopack Productos Químicos, Zárate, BA, Arg]; 10 mM Na_2_HPO_4_ [cat. 2000163900, Biopack Productos Químicos, Zárate, BA, Arg]; 1.8 mM KH_2_PO_4_ [cat. 2000168907, Biopack Productos Químicos, Zárate, BA, Arg]; pH 7.4), and resuspended in supplemented α-minimal essential medium (α-MEM; cat. 11900024, Gibco, Grand Island, NY, USA) containing 2 mM L-glutamine (cat. 25030081, Gibco, Grand Island, NY, USA), 100 IU/mL antibiotic-antimycotic (cat.15240062, Gibco, Grand Island, NY, USA), and 20% heat-inactivated fetal bovine serum (FBS; cat.16000044, Gibco, Grand Island, NY, USA) [Sup-α-MEM].

Total MNC number was determined using a 3% acetic acid solution and cell viability was assessed by 0.04% trypan blue dye exclusion. Viable MNCs (10 × 10^6^) from BM samples of HVs and BCPs were seeded into 25 cm^2^ tissue culture flasks containing 10 mL of Sup-α-MEM and maintained at 37°C in a humidified atmosphere with 5% CO_2_. After 24 h, non-adherent cells were removed and fresh medium was added. Primary cultures were expanded until reaching 75%–80% sub-confluence, with medium replacement every 7 days.

Stromal cells from one first-passage flask were washed twice with PBS, detached using a 0.05% trypsin/EDTA solution in PBS (cat. 15400054, Gibco, Grand Island, NY, USA), and replated in two new 25 cm^2^ flasks. Cells were allowed to proliferate until reaching 75%–80% confluence again. Following the second passage, stromal cells were plated at low density (240 cells/cm^2^) in 25 cm^2^ flasks and cultured in Sup-α-MEM for 12 days, with medium renewal on day 6. This low-density plating strategy favors enrichment of MSCs with high self-renewal and multipotency, as described by Ylöstalo et al. [30].

Surface phenotype analysis was performed to confirm that the cells from the 3rd subculture met the minimal criteria for defining MSCs established by The International Society for Cellular Therapy [31]. After this period, cells were washed twice with PBS, trypsinized, washed again, and replated in 25 cm^2^ flasks at a density of 3000 viable cells/cm^2^. Cells were cultured under the conditions described above, with medium renewal every 7 days until reaching 75%–80% confluence (4th subculture). Subsequently, MSCs were washed twice with PBS, trypsinized, counted, and used for various assays. This change in plating density was intended to increase the number of MSCs available for different assays. Importantly, this change in deeding density does not affect the characteristics of MSCs, as the same density is used in osteogenic and adipogenic plasticity assays [21,32].

### 2.3 Study of Bone Marrow Infiltration with Neoplastic Cells

BM infiltration by neoplastic cells was evaluated by immunocytochemistry in combination with morphological assessment. The aspirates were processed using an immunocytochemistry detection system (cat. K0673, Dako, Carpinteria, CA, USA) and cell morphology was evaluated using the Pappenheim technique. For epithelial markers detection, samples were incubated with antibodies (Abs) directed against cytokeratins AE1–AE3 (cat. IR053, Dako, Carpinteria, CA, USA) and epithelial membrane antigen (EMA; cat. M0613, Dako, Carpinteria, CA, USA). Samples were classified as positive for metastasis only when cells exhibited both cytokeratin AE1–AE3 and EMA expression together with malignant morphological features. Isotype control Abs (cat. 08-6599, ZYMED Laboratories, South San Francisco, CA, USA, and cat. X0943, Dako, Carpinteria, CA, USA) were included in parallel, using the same antibody concentrations as the corresponding primary antibodies. Each experiment was performed twice for every sample.

### 2.4 Phenotypic Characterization of Mesenchymal Stem/ Stromal Cells

MSCs from the 3rd subculture were suspended in PBS containing 1% bovine serum albumin (BSA; cat. A7030, Sigma Aldrich, St. Louis, MO, USA) and incubated with fluorochrome-conjugated primary Abs directed against the following human surface markers: CD105 (cat. FAB10971V, R&D Systems Inc., Minneapolis, MN, USA), CD90 (cat. FAB2067G, R&D Systems Inc., Minneapolis, MN, USA), CD73 (cat. FAB5795A, R&D Systems Inc., Minneapolis, MN, USA), CD79a (cat. FAB69201G, R&D Systems Inc., Minneapolis, MN, USA), CD11b (cat. FAB1699V, R&D Systems Inc., Minneapolis, MN, USA), CD34 (cat. FAB7227P, R&D Systems Inc., Minneapolis, MN, USA) and C-C motif chemokine receptor 2 (CCR2) (cat. FAB151P, R&D Systems Inc., Minneapolis, MN, USA). Corresponding isotype-matched control Abs (cat. IC002V, IC003G, IC0041A, IC002G, IC002V, IC002P, IC0041P, R&D Systems Inc., Minneapolis, MN, USA) were processed in parallel at identical concentrations.

After incubation for 30 min at room temperature, samples were acquired on a FACScanto II flow cytometer (BD Biosciences, San Diego, CA, USA). A minimum of 10,000 events per sample were recorded and analyzed relative to the corresponding isotype controls. Data analysis and histogram generation were performed using FlowJo software (v. X, Tree Star Inc., Ashland, OR, USA). All experiments were conducted in duplicate using independent MSC preparations.

### 2.5 Quantitative Real-Time PCR

Total ribonucleic acid (RNA) was extracted from MSCs of the 4th subculture (BCPs, n = 10 and HVs, n = 10) using TRI Reagent^®^ (cat. TR 118, Molecular Research Center Inc., Cincinnati, OH, USA). Reverse transcription was carried out using 1 µg RNA and random primers (cat. 4368814, Applied Biosystems, Foster City, CA, USA) to generate copy DNA (cDNA). Quantitative PCR was performed in duplicate using SYBR Green master mix (cat. 04913850001, ROCHE, Mannheim, Germany) on a real-time PCR system (CFX96™ TOUCH REAL-TIME PCR, Bio-Rad, Hercules, CA, USA) under standard cycling conditions. Amplification specificity was verified by melting curve analysis. The threshold cycle (Ct) values were normalized against the reference gene glyceraldehyde-3-phosphate dehydrogenase (GAPDH) and data are presented as the fold change in gene expression, relative to the HVs group. Primer sequences are provided in Table 1.

**Table 1:** DNA primer sequences.

Primer	Sequence (5^′^-3^′^)	Size (bp)
HSP90-forward	AAGTCTGGGACCAAAGCGTTC	218
HSP90-reverse	GTTCCACGACCCATAGGTTCAC	
Nrf2forward	GCTATGGAGACACACTACTTGG	211
Nrf2reverse	CCAGGACTTACAGGCAATTCT	
SOD1forward	GGTCCTCACTTTAATCCTCTAT	97
SOD1reverse	CATCTTTGTCAGCAGTCACATT	
SOD2forward	TGACAAGTTTAAGGAGAAGC	149
SOD2reverse	GAATAAGGCCTGTTGTTCC	
GPx1forward	CGCCACCGCGCTTATGACCG	238
GPx1reverse	GCAGCACTGCAACTGCCAAGCAG	
CATforward	CTCAGGTGCGGGCATTCT	69
CATreverse	CAATGTTCTCACACAGACGTTTCC	
GAPDH-forward	CCACATCGCTCAGACACCAT	178
GAPDH-reverse	CATGGGTGGAATCATATTGGA	

Note: bp: base pairs.

### 2.6 A Single-Cell Gel Electrophoresis Assay (Comet Assay)

To detect DNA damage, the comet assay was performed under alkaline conditions, following the protocol described in Collins et al. [33]. Briefly, 2 × 10^4^ MSCs (BCPs, n = 7 and HVs, n = 5) from the 4th subculture were resuspended in 75 μL of 1% low-melting-point (LMP) agarose (cat. V2111, Promega, Madison, WI, USA) solution and seeded onto a pre-treated glass slide (cat. D100002, Eurotubo Deltalab SL, Barcelona, Spain) with 1% normal-melting-point (NMP) agarose (cat. A4718, Sigma Aldrich, St. Louis, MO, USA). Subsequently, a third layer of LMP agarose was applied. The seeded slides were placed in a cold lysis solution (0.5 M NaCl, 100 mM EDTA [cat. E5134, Sigma Aldrich, St. Louis, MO, USA], 10 mM Tris [cat. 2000166800, Biopack Productos Químicos, Zárate, BA, Arg.], pH 10, 1% Triton X-100 [cat. X100, Sigma Aldrich, St. Louis, MO, USA]) and maintained at 4°C for at least 18 h. After this period, they were stabilized in PBS at 4°C for 1 h. Immediately after, the slides were transferred to the electrophoretic tank containing alkaline solution (300 mM NaOH [cat. 1223, Laboratorios Cicarelli, San Lorenzo, SF, Arg], 1 mM EDTA, pH 13) and kept for 20 min at 4°C in the dark. For the electrophoresis run, a current of 20 V (constant voltage) was applied for 30 min in the dark. The slides were then removed and incubated in neutralization solution (0.4 M Tris, pH 7.5) for 5 min and stained with 20 μL of ethidium bromide (20 μg/mL; cat. H5041, Promega, Madison, WI, USA). The nucleoids (cytoplasmic area containing the cell’s genetic material) were evaluated using a fluorescence microscope. A total of 100 comets per sample were randomly scored based on the intensity of fluorescence, tail length, and nucleoid integrity. The comets were classified into 5 categories, ranging from C0 (Class 0, no DNA damage, no detectable tail) to C4 (Class 4, high level of DNA damage, almost all DNA in the comet tail). Based on this classification, a score was calculated according to the following formula: score [arbitrary units] = nC1 + nC2 **·** 2 + nC3 **·** 3 + nC4 **·** 4, resulting in values between 0 and 400, where n is the number of comets counted in each category.

Untreated human umbilical cord MSCs (HUC-MSCs) were used as negative controls, and 0.1 mM H_2_O_2_-treated HUC-MSCs were used as positive controls (kindly donated by Dr. Esteban Fiore, Instituto de Investigaciones en Medicina Traslacional-CONICET, Facultad de Ciencias Biomédicas, Universidad Austral, Provincia de Buenos Aires, Argentina) [33]. The experiments were repeated twice for each sample.

### 2.7 Chromosomal Aberrations Analysis

The methodology used was described by de Campos-Nebel et al. [34]. MSCs (BCPs, n = 5 and HVs, n = 3) from the 3rd subculture were seeded at a density of 3000 cells/cm^2^ in 25 cm^2^ flasks (4th subculture) and cultured in Sup-α-MEM until reaching 50% confluence (exponential growth phase). Subsequently, the culture medium was changed to Sup-α-MEM containing, in this opportunity, 30% FBS and 0.04 μg/mL of colcemid (N-desacetyl-N-methylcolchicine; cat. 15212012, Gibco, Grand Island, NY, USA) for 18 h. Afterwards, cells were washed twice with PBS and treated with a solution of 0.05% trypsin-0.02% EDTA (cat. 15400054, Gibco, Grand Island, NY, USA). To the cell suspension, 10% FBS was added and centrifuged at 448× *g* for 10 min. Cells were re-suspended in a hypotonic solution of 75 mM KCl for 35 min, fixed in a mixture of methanol (cat. 2000160900, Biopack Productos Químicos, Zárate, BA, Arg) and acetic acid (3:1; cat. 1025110, Laboratorios Cicarelli, San Lorenzo, SF, Arg) for 24 h at −20°C, dropped onto slides, and stained with 10% Giemsa (cat. 2000110000, Biopack Productos Químicos, Zárate, BA, Arg) in water for 4 min. Fifty metaphase cells were analyzed per sample to determine the number of CAs under a bright-field microscope with a 100× objective (CX31, Olympus, Tokyo, Japan). Any metaphase cell presenting at least one CA was considered abnormal. The CAs were classified as chromatid breaks (CB), acentric fragments (AF) and chromatid exchanges [telomeric associations (TAS), ring chromosomes (RC), and dicentric chromosomes (DIC)] [35].

### 2.8 Quantification of Nuclear Blebs and Micronuclei

MSCs (BCPs, n = 6 and HVs, n = 6) from the 3rd subculture were seeded at a density of 3000 cells/cm^2^ on glass coverslips in 6-well plates (cat. 4430300, Orange Scientific, Braine-l’Alleud, Belgium) and cultured in Sup-α-MEM until reaching 50% confluence (4th subculture). After this period, the culture was washed twice with PBS and fixed with a 4% paraformaldehyde (PFA) solution in PBS for 15 min at room temperature. They were then stained with pure Giemsa for 5 min, mounted and analyzed under a bright-field microscope with a 100× objective (Eclipse E200, Nikon Instruments Inc., Tokyo, Japan). The frequency of NBs and MN was evaluated per 1000 cells [36].

### 2.9 Oxidative Damage to Proteins

Protein carbonyls are a general marker of protein oxidation generated by various radicals. The experiments were conducted with protein lysates from MSCs (BCPs, n = 4 and HVs, n = 5) of the 4th subculture. Viable cells (1 × 10^5^) were resuspended in 100 μL of Thermo Scientific™ NP-40 lysis buffer (cat. J60766.AP, Thermo Scientific™, Waltham, MA, USA) supplemented with 1× Complete™ Protease Inhibitor Cocktail (cat. 11873580001, Roche, Mannheim, Germany). The lysates were incubated on ice for 30 min and then centrifuged at 10,000× *g* for 20 min at 4°C. The supernatant was collected, and protein concentrations were measured using the DC Protein Assay (cat. 5000111, Bio-Rad, Hercules, CA, USA). Carbonyl concentrations can be assessed via their reaction with 2,4-dinitrophenylhydrazine (DNPH; cat. D199303, Sigma Aldrich, St. Louis, MO, USA) to form hydrazone, a yellow product that can be quantified spectrophotometrically. To quantify total protein carbonyl concentrations, the protocol described by Hawkins et al. “*Quantification of protein carbonyls: Total carbonyls on proteins*” was followed [37]. Protein oxidation was also assessed in the conditioned medium of MSCs (secretome). For this assay, once reaching 75%–80% confluence at the 4th subculture, some of the cell cultures were washed twice with PBS and incubated at 37°C, 5% CO_2_, and high humidity for 48 h in α-MEM with 2 mM L-glutamine, 100 IU/mL antibiotic/antimycotic, but without FBS supplementation. After this period, the conditioned media (CM) were collected, centrifuged at 250× *g* for 10 min, aliquoted, and stored at −80°C until evaluation of carbonyl protein. All measurements were performed in duplicate.

### 2.10 Oxidative Damage to Lipids

Lipid peroxidation was indirectly assessed through the study of the presence of 4-hydroxynonenal (4-HNE) protein adducts in protein lysates from MSCs (BCPs, n = 4 and HVs, n = 3) of the 4th subculture. The 4-HNE protein adducts refer to the chemical modifications that occur in proteins due to the binding with this byproduct. 4-HNE is a product of lipid peroxidation, a process in which lipids present in cell membranes are damaged by ROS, producing reactive compounds [38,39]. Protein lysates were obtained from viable cells following the protocol previously described. A total of 25 µg of protein per sample was resolved in a 12% sodium dodecyl sulfate-polyacrylamide (SDS-PAGE) gel. The resolved protein bands were electroblotted onto a polyvinylidene fluoride membrane (PVDF; cat. 1620177, Bio-Rad, Hercules, CA, USA). Molecular weights were determined using the PageRuler™ (cat. 26616, Thermo Scientific™, Vilnius, LT, Lithuania). Membranes were then blocked for 45 min in 1× Tris-buffered saline (TBS; 50 mM Tris-HCl [cat. H5123, Promega, Madison, WI, USA]; 150 mM NaCl; pH 7.4) containing 0.1% TWEEN 20 (T-20) and 5% (w/v) non-fat dried milk, and subsequently incubated overnight at 4^°^C with primary Abs against 4-HNE monoclonal Abs (cat. Ab243070, ABCAM, Cambridge, MA, USA) and β-actin (cat. A5441, Sigma Aldrich, St. Louis, MO, USA), used as a loading control. Subsequently, the membranes were washed with washing buffer (0.01% T-20 in TBS) and incubated with the horseradish peroxidase (HRP)-conjugated secondary Abs (cat. 170-6516, BIORAD, Hercules, California, USA) for 1 h at room temperature. Finally, the membranes were washed three times with washing buffer and the bound Abs were detected using the chemiluminescent detection system (cat. 1705060, BioRad, Hercules, California, USA) according to the manufacturer’s instructions and visualized by exposure to photographic films. The developed films were subsequently digitized using the ChemiDoc MP Imaging System (BIORAD, Hercules, CA, USA). The bands were quantified by densitometry using ImageJ (V 1.5.4, National Institutes of Health, Bethesda, MD, USA). The density of the protein bands was normalized to β-actin expression.

### 2.11 Collection of Conditioned Media for Proteomic Analysis

After the collection and storage of the CM, 10 mL of each secretome were taken and pooled into BCPs-CM (BCPs, n = 6) and HVs-CM (HVs, n = 6) according to their respective groups. The total 60 mL from each study group was then lyophilized for 48 h in a laboratory freeze-drying system, following the manufacturer’s instructions (Modulyod 115, Thermo Fisher, Carlsbad, CA, USA). The lyophilized samples were reconstituted in 4 mL of sterile H_2_O (cat. 956-A, Rivero, CABA, Arg). The corresponding concentrated media (~15-fold) from each study group was used for proteomic analysis. Protein concentration was determined using the Bradford method with BSA (cat. sc-2323, Santa Cruz, CA, USA) as the standard [40].

### 2.12 Protein Content Analysis of Conditioned Media (Secretome) from Mesenchymal Stem/Stromal Cells

Thirty micrograms of proteins per lane were subjected to 1D electrophoresis on a 10% SDS-polyacrylamide gel under reducing conditions, with each sample (BCPs-CM and HVs-CM) loaded in triplicate. Electrophoresis was carried out at a constant voltage of 80 V until the front reached 1 cm below the stacking gel.

Immediately after electrophoresis, triplicates of each sample were fixed with fixing buffer (30% v/v ethanol [cat. E7023, Sigma Aldrich, St. Louis, MO, USA] and 2% v/v phosphoric acid [cat. 93752, Sigma Aldrich, St. Louis, MO, USA] in H_2_O) with agitation overnight at room temperature.

After fixation, gels were washed three times for 30 min with H_2_O and equilibrated in equilibration buffer (18% v/v methanol, 17% w/v ammonium sulfate (cat. AX1385, Sigma Aldrich, St. Louis, MO, USA), and 2% v/v phosphoric acid in sterile H_2_O) with agitation for 1 h at room temperature. Subsequently, Coomassie Brilliant Blue G-250 (cat. 1610406, BioRad, Hercules, California, USA) was added to the buffer by sprinkling, reaching a final concentration of 0.5 g/L (staining solution). The gel was incubated in this solution overnight with agitation at room temperature. The next day, the gel was washed three times for 30 min with H_2_O, and protein bands were excised from the polyacrylamide gel using a sterile scalpel and placed in individual tubes for further analysis. Proteomic analysis was conducted by the Centro de Estudios Químicos y Biológicos por Espectrometría de Masa (CEQUIBIEM) at the Facultad de Ciencias Exactas y Naturales, Universidad de Buenos Aires, using the label-free protein quantification (LFQ) proteomic method. Briefly, the samples were reduced with 20 mM Ditiotreitol (DTT; cat. 43816, Sigma Aldrich, St. Louis, MO, USA) for 45 min at 56°C and alkylated with 50 mM iodoacetamide (cat. I1149, Sigma Aldrich, St. Louis, MO, USA) for 45 min in the dark. They were then digested with trypsin (cat. T7575, Sigma Aldrich, St. Louis, MO, USA) overnight. Peptides were extracted using acetonitrile (cat. 100029; Merck, Darmstadt, Germany). The samples were lyophilized using a Speed Vac (Thermo Fisher Scientific, Waltham, MA, USA) and then resuspended in 30 µL of 0.1% trifluoroacetic acid (cat. 80457, Sigma Aldrich, St. Louis, MO, USA). Desalting was performed using a ZipTip C18 (cat. ZTC18S096, Merck Millipore Ltd., Germany) and separated by nanoLC (EASY-nLC 1000, Thermo Fisher Scientific, Waltham, MA, USA) before being analyzed by tandem mass spectrometry (spectrometer Q-Exactive, Thermo Fisher Scientific, Waltham, MA, USA) with Orbitrap technology (nano HPLC-ESI-MS/MS). A voltage of 1.5–3.5 kV was used for ElectroSpray Ionization (EASY-SPRAY, Thermo Fisher Scientific, Waltham, MA, USA). The MS equipment features a high collision dissociation cell (HCD) for fragmentation and an Orbitrap analyzer (Q-Exactive, Thermo Fisher Scientific, Waltham, MA, USA). XCalibur software (V 3.0.63, Thermo Fisher Scientific, Waltham, MA, USA) was used for data acquisition. Raw spectra were analyzed using Proteome Discoverer software (version 2.2, Thermo Fisher Scientific, Waltham, MA, USA) for protein identification. Spectra were searched against the UniProt proteome reference from the *Homo sapiens* database UP000005640 using standard settings. Statistically significant differences in relative abundances between the two analyzed groups (BCPs-CM and HVs-CM) were assessed using Perseus software (V 2.0.11, available at https://maxquant.net/perseus/). Differentially expressed proteins (DEPs) in BCPs and HVs were identified using a two-sided Student’s t-test in combination with a permutation-based false discovery rate correction (FDR).

To gain molecular insight into the DEPs identified, a Gene Ontology (GO) enrichment analysis was performed for biological processes using the Database for Annotation, Visualization, and Integrated Discovery (DAVID; v2023q4, NIH, Bethesda, MD, USA) [41], applying an EASE threshold of 0.05 and a Count threshold of 2 in all cases.

### 2.13 Statistical Tests

Results are given as the mean ± standard error (SE) when appropriate. Data normality was assessed using the Shapiro–Wilk test. For comparisons between two groups, an unpaired Student’s *t*-test with Welch’s correction was applied when data followed a normal distribution, whereas the Mann–Whitney U test was used for non-normally distributed variables. Categorical data were analyzed using the Chi-square test when expected frequencies met test assumptions. For proteomic analyses, a permutation-based FDR = 0.05 correction was used. Differences were considered statistically significant when *p* < 0.05.

## 3 Results

### 3.1 Study of Bone Marrow Infiltration with Neoplastic Cells

Bone marrow aspirates from the posterior iliac crest showed no evidence of infiltration by neoplastic cells in any of the cancer patients studied.

### 3.2 Mesenchymal Stem/ Stromal Cell Phenotypic Characterization

**Figure 1:**
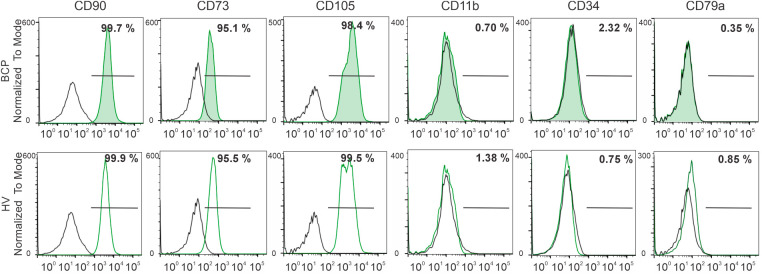
Phenotypic characterization of bone marrow-derived mesenchymal stem/ stromal cells (BM-MSCs). Representative flow cytometry histograms of MSC surface antigens from a breast cancer patient (BCP) and healthy volunteer (HV). Isotype controls are shown as open black histograms, whereas specific surface antigen staining is shown in green histograms (shaded in BCP samples and open in HV samples). The percentage of positive cells for each marker is indicated. CD: Cluster of Differentiation.

MSCs at the third passage were phenotypically characterized by flow cytometry using classic surface markers. Over 95% of MSCs from both groups (BCPs, n = 10 and HVs, n = 10) expressed CD73, CD105, and CD90, while showing no expression of CD11b, CD34, or CD79a (Fig. 1). Thus, the MSCs employed in this study meet the minimal criteria established for defining MSCs [31].

### 3.3 Expression of CCR2 in Mesenchymal Stem/Stromal Cells

**Figure 2:**
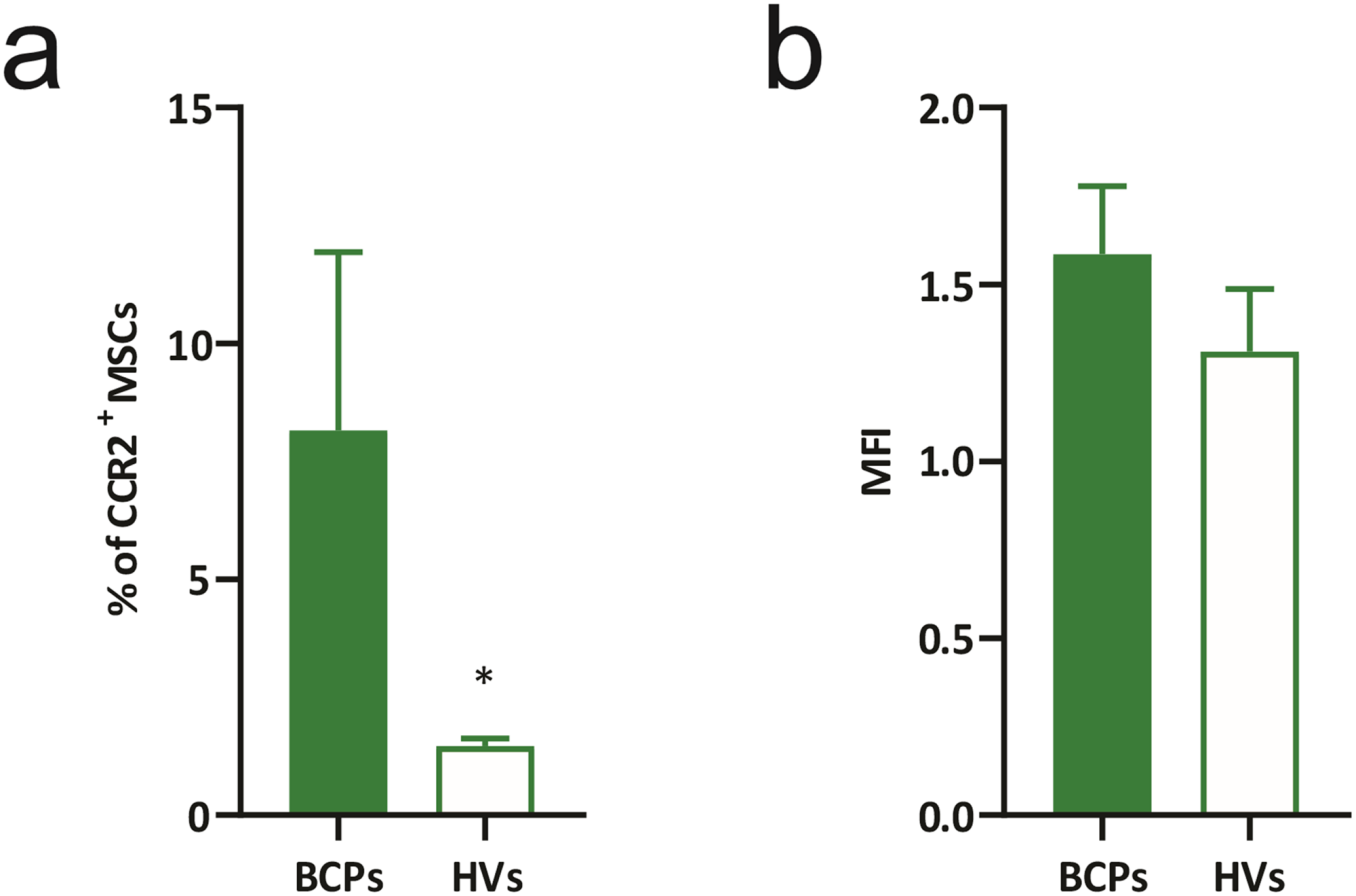
Expression of CCR2 in mesenchymal stem/ stromal cells (MSCs). (**a**) Percentage of CCR2^+^ MSCs from breast cancer patients (BCPs, n = 6) and healthy volunteers (HVs, n = 6). Values are expressed as mean ± standard error. Statistical analysis: Mann–Whitney U test. Asterisks indicate a significant difference (*p < 0.05). (**b**) Relative fluorescence intensity of CCR2 in MSCs from BCPs (n = 6) and HVs (n = 6). Median fluorescence intensity (MFI), values were normalized to the HV group. Values are expressed as mean ± standard error. CCR2: C-C Motif Chemokine Receptor 2.

It has been reported that soluble factors such as CCL-2, via CCR2, drive MSC senescence through ROS [42]. These observations led us to investigate CCR2 expression in MSCs from BCPs, as previous studies showed elevated CCL-2 levels in their conditioned media compared with HVs [20]. Thus, the CCL2/CCR2 interaction may enhance ROS accumulation, thereby promoting oxidative modifications of lipids, proteins, and DNA. Notably, although only a small subset of BM-MSCs expressed CCR2, the proportion of CCR2^+^ cells was significantly higher in BCPs than in HVs (Fig. 2a), while the mean fluorescence intensity of CCR2^+^ cells remained comparable between groups (Fig. 2b), indicating that the difference arises from a higher frequency of positive cells rather than from increased receptor density per cell.

### 3.4 Increased Expression of Oxidative Stress Response Genes in Mesenchymal Stem/ Stromal Cells from Breast Cancer Patients

**Figure 3:**
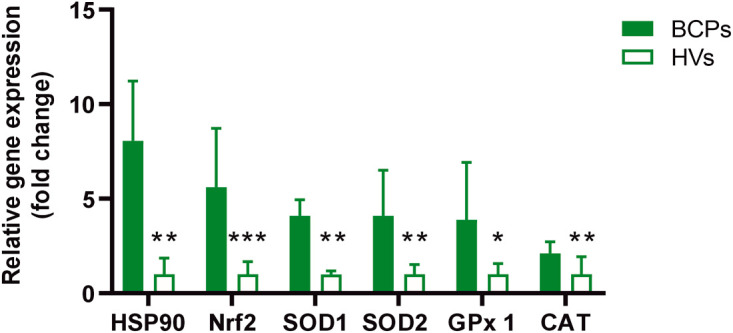
Expression of oxidative stress response factors in bone marrow mesenchymal stem/ stromal cells (MSCs). Samples of MSCs were obtained from breast cancer patient (BCPs, n = 10) and healthy volunteers (HVs, n= 10). The expression of specific genes (HSP90, Nrf2, SOD1, SOD2, GPx1, and CAT) was quantitative by real-time polymerase chain reaction (PCR) assay. All results were normalized to a reference gene GAPDH and are presented relative to the expression level in MSCs from HVs. Values are expressed as mean ± standard error. Statistical analysis: unpaired t-test with Welch’s correction. Asterisks indicate a significant difference (*p < 0.05, **p < 0.01 and ***p < 0.001). HSP90: heat shock protein 90 alpha; Nrf2: nuclear factor erythroid 2-related factor 2; SOD1: superoxide dismutase 1; SOD2: superoxide dismutase 2; GPx1: glutathione peroxidase 1; CAT: catalase.

Our recent studies demonstrated that BM-MSCs from this patient group exhibit increased oxidative stress [16]. In this regard, we assessed the expression of genes related to oxidative stress response, such as HSP90 and Nrf2, as well as antioxidant response genes, including SOD1, SOD2, CAT, and GPx1. Our findings revealed that MSCs from BCPs showed overexpression of both oxidative stress-related genes and antioxidant response genes (Fig. 3).

### 3.5 Enhanced Oxidative Damage in Mesenchymal Stem/ Stromal Cells from Breast Cancer Patients

#### 3.5.1 DNA Damage, Comet Assay

**Figure 4:**
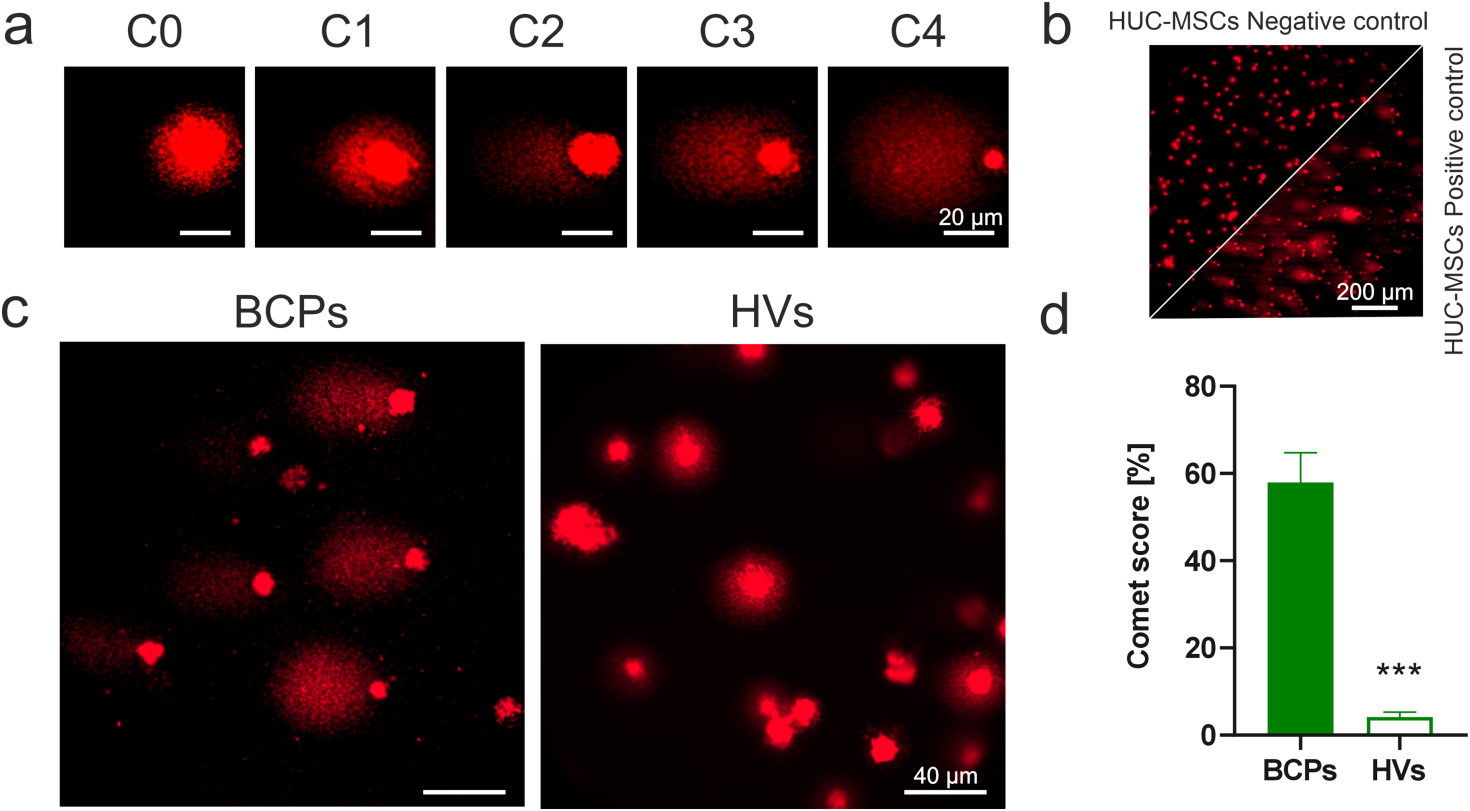
DNA oxidative damage in mesenchymal stem/stromal cells (MSCs) from bone marrow (BM) in breast cancer patients (BCPs). (**a**) BM-MSCs from BCPs processed using comet assay and stained with Ethidium Bromide and analyzed by fluorescence microscopy. Comets were classified into five categories (C0–C4). (**b**) Technical controls: untreated human umbilical cord MSCs (HUC-MSCs; negative control, left) and HUC-MSCs treated with 0.1 mM H_2_O_2_ (positive control, right). (**c**) Representative panel, MSCs from a BCP and a healthy volunteer (HV). (**d**) Comet score expressed as a percentage of BCPs (n = 7) and HVs (n = 5). Values are expressed as mean ± standard error. Statistical analysis: unpaired t-test with Welch’s correction. Asterisk indicates a significant difference (***p < 0.001).

The comet assay was used to evaluate DNA damage in MSCs from BCPs and HVs. In this technique, damaged DNA migrates from the cell nucleus in a manner resembling the tail of a comet [43]. The more substantial the DNA breakage/damage, the more pronounced the comet tail will be (Fig. 4a). Positive and negative technical controls were performed in parallel using HUC-MSCs treated or not with 0.1 mM H_2_O_2_ (Fig. 4b). The results of this assay show that MSCs from BCPs form comets with morphologies more associated with DNA damage compared to those from HV samples (Fig. 4c). The comet score calculation revealed an increase for BCPs, indicating a higher average DNA damage in MSCs from this group (Fig. 4d).

#### 3.5.2 Breast Cancer Patients Exhibit a Higher Proportion of Bone Marrow Mesenchymal Stem/ Stromal Cells with Chromosomal Aberrations, Nuclear Blebs, and Micronuclei

**Figure 5:**
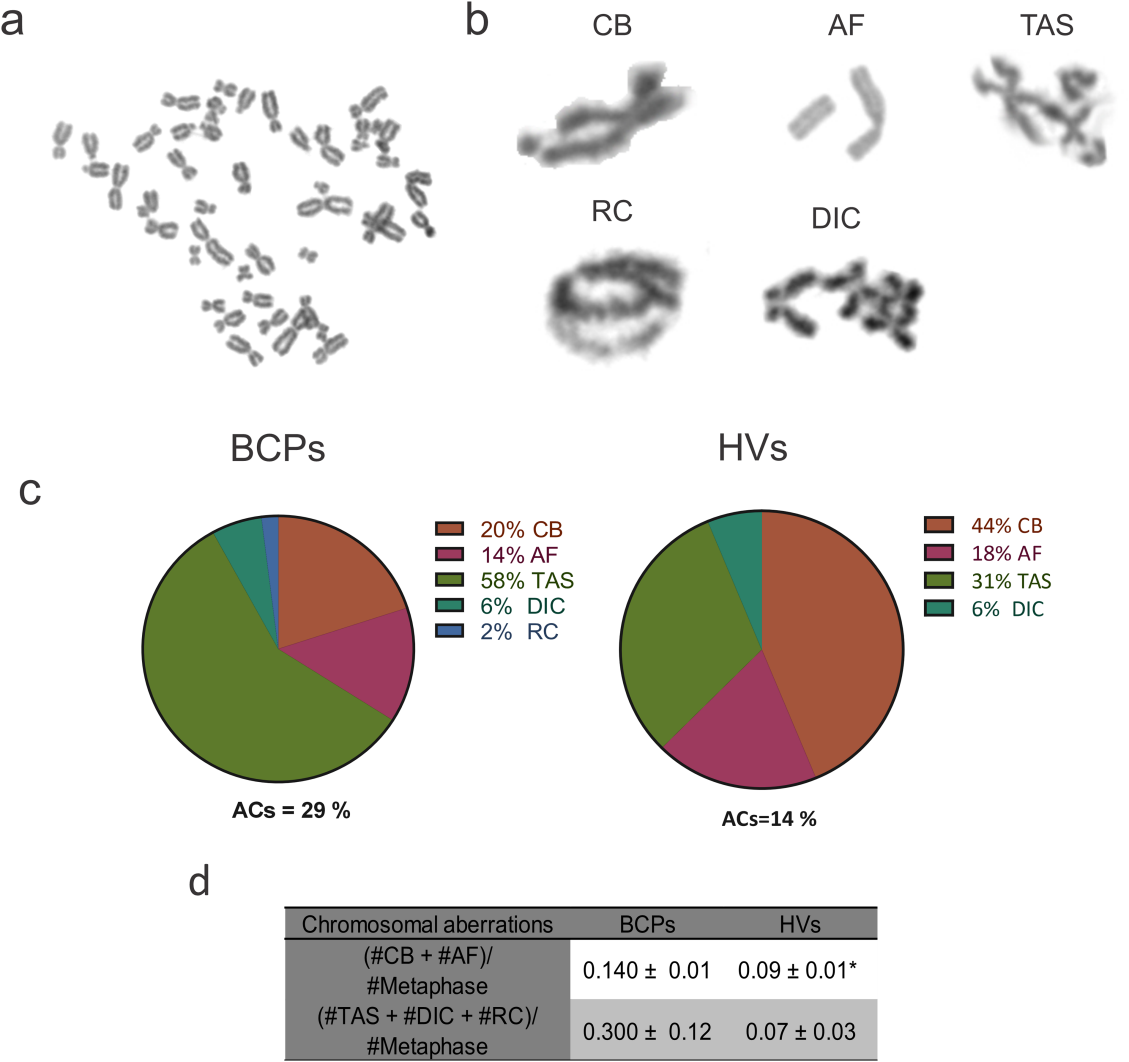
Structural chromosomal aberrations observed in mesenchymal stem/stromal cells (MSCs) from breast cancer patients (BCPs). (**a**) Normal metaphase (without chromosomal aberrations) in a BCP, 2n = 46, XX. (**b**) Examples of observed structural chromosomal aberrations: (i) Chromatid break (CB), (ii) Acentric fragment (AF), (iii) Telomeric association (TAS), (iv) Ring chromosome (RC) and (v) Dicentric chromosome (DIC). (**c**) Diagram of the percentage distribution of structural chromosomal aberrations in abnormal MSCs with chromosomal aberrations in metaphase in BCPs (n = 5) and healthy volunteers (HVs, n = 3). ACs: abnormal cells. (**d**) Clustered chromosomal aberration analysis. Values are expressed as mean ± standard error. Statistical analysis: Chi-square test. Asterisks indicate a significant difference (*p < 0.05).

ROS can cause DNA strand breaks and other types of damage, which, if not properly repaired, can lead to chromosomal aberrations (Fig. 5a,b). When evaluating the number of abnormal cells, defined as the number of metaphases with at least one CA out of the total metaphase cells assessed, we found a trend towards an increased number of abnormal MSCs in the BCPs group compared to the HVs group (Fig. 5c).

Furthermore, when analyzing the frequency of aberrations categorized by the number of breaks required for their occurrence—one break for CB and AF, and two breaks for TAS, RC, and DIC—we observed that the frequencies were significantly higher for the BCPs compared to the HVs (Fig. 5c,d).

**Figure 6:**
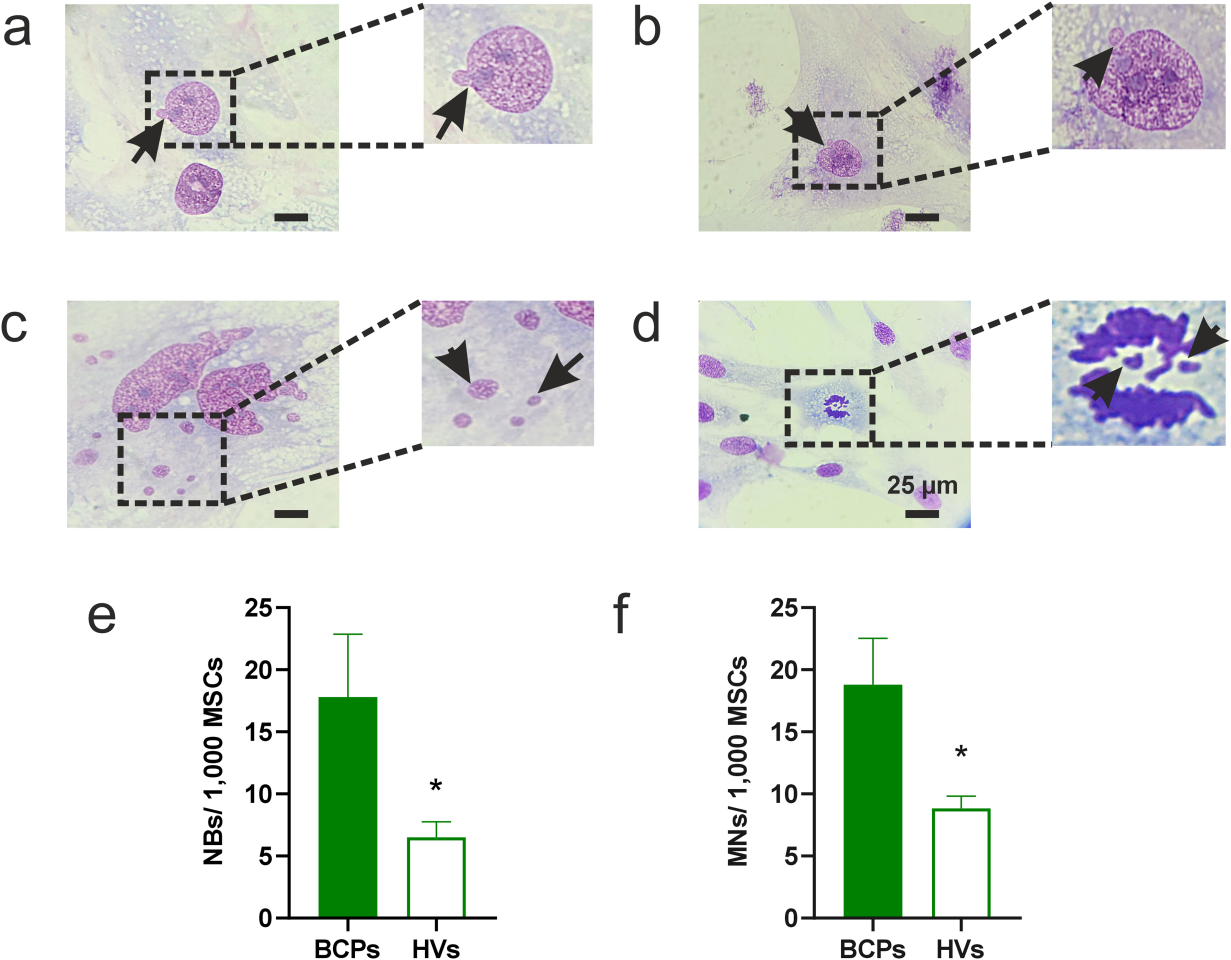
Frequency of mesenchymal stem/stromal cells (MSCs) with nuclear blebs (NBs) and micronuclei (MN) in breast cancer patients (BCPs). (**a**) NBs. The arrow indicates the NBs observed in an MSC from a BCP. (**b**) MN. The arrow indicates the MN observed in an MSC from a BCP. (**c**) Multimicro-nucleated MSC from a BCP. Arrows show the multiple MN. (**d**) Anaphase lag in MSC from a BCP under standard culture conditions. Arrows indicate lagging chromosomes, which lead to the formation of MN. Microphotographs were taken at a total magnification of 1000×, localized zoom 2×. (**e**) Differences observed for NBs in the BCPs (n = 6) and healthy volunteers (HVs, n = 6). (**f**) Differences observed for MN in the BCPs (n = 6) and HVs (n = 6). Values are expressed as mean ± standard error. Statistical analysis: unpaired t-test. Asterisk indicates a significant difference (*p < 0.05).

Another indicator of damage and oxidative stress is the formation of NBs and/or MN. In this regard, we found NBs and MN in all MSC samples observed from both BCP and HV groups (Fig. 6a,b). It is noteworthy that multinucleated cells and metaphase cells with lagging chromosomes were also identified (Fig. 6c,d). Moreover, when evaluating the percentage of MSCs with NBs and MN, a higher percentage was observed in the BCPs group compared to the HVs group (Fig. 6e,f).

#### 3.5.3 Protein and Lipid Damage

**Figure 7:**
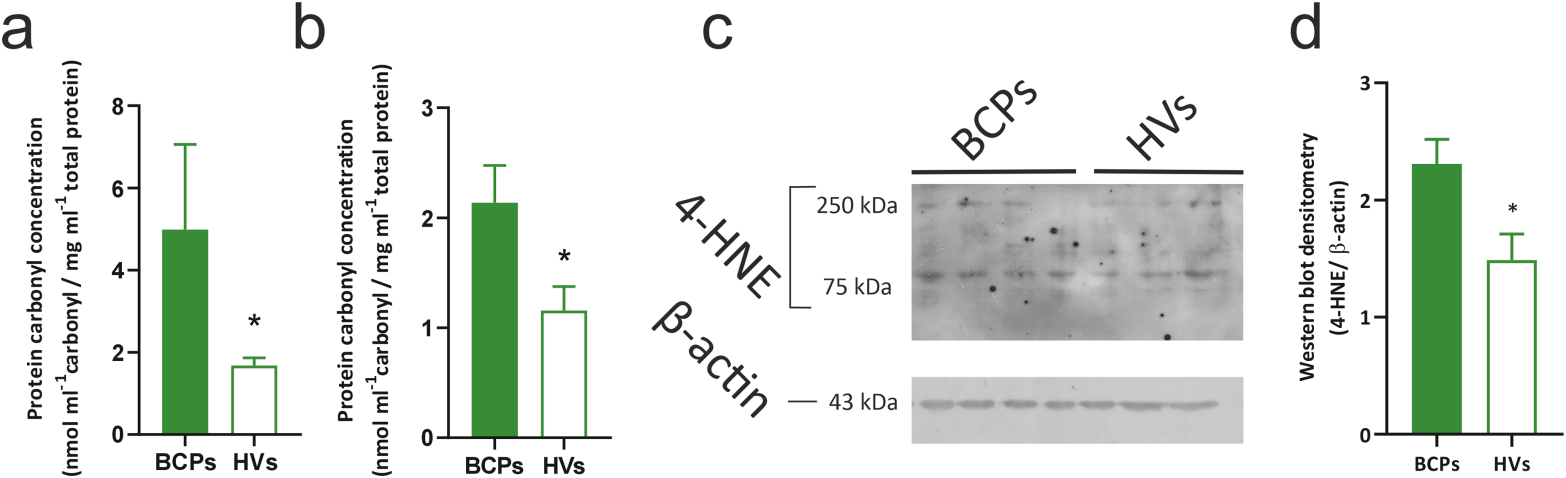
Oxidative damage to proteins and lipids in bone marrow-derived mesenchymal stem/ stromal cells (MSCs) from breast cancer patients (BCPs) and conditioned media from BCPs and healthy volunteers (HVs). (**a**) Carbonylated protein content in protein extracts from MSCs of BCPs (n = 4) and HVs (n = 5). Values are expressed as mean ± standard error (SE). (**b**) Carbonylated protein content in conditioned media from MSCs of BCPs (n = 4) and HVs, (n = 5). Values are expressed as mean ± SE. (**c**) Immunoblot of bone marrow-derived MSC proteins extracts from BCPs and HVs, using antibodies against 4-hidroxinonenal (4-HNE) and β-actin. (**d**) Abundance of 4-HNE protein in MSC protein extracts from BCPs (n = 4) and HVs (n = 3). The expression of 4-HNE and β-actin was analyzed by immunoblotting, and the images were analyzed by densitometry. Values are expressed as mean ± SE. Statistical analysis: unpaired t-test with Welch’s correction. Asterisks indicate a significant difference (*p < 0.05).

Interestingly, and in accordance with the previous results, when evaluating oxidative damage in protein extracts of MSCs, increased values of carbonylated proteins were found in the BCPs group compared to the HVs group (Fig. 7a). At the same time, the assessment of protein oxidation in the CM of MSCs (Secretome) revealed an increase in carbonylated proteins for the BCPs group (Fig. 7b). Regarding oxidative damage in lipids, it was assessed through the presence of 4-HNE, a byproduct of lipid peroxidation formed when lipids in cell membranes undergo oxidative damage. SDS-PAGE analysis revealed an increase in the abundance of adducts in a subset of proteins of various sizes, ranging from approximately 250 to 75 kDa, in MSCs from BCPs compared to the HVs (Fig. 7c,d).

#### 3.5.4 Characterization of the Secretome of Mesenchymal Stem/Stromal Cells from Breast Cancer Patients

It is well known that MSCs release a variety of paracrine factors, including cytokines, growth factors, and extracellular vesicles, which influence the surrounding microenvironment. The study of the secretome of BM-MSCs in the PMN context of these BCPs is crucial, as the factors they release play a key role in creating an optimal environment for extravasation and interaction with circulating cancer cells.

**Figure 8:**
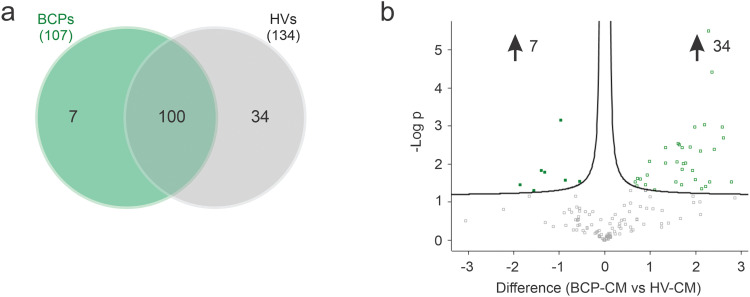

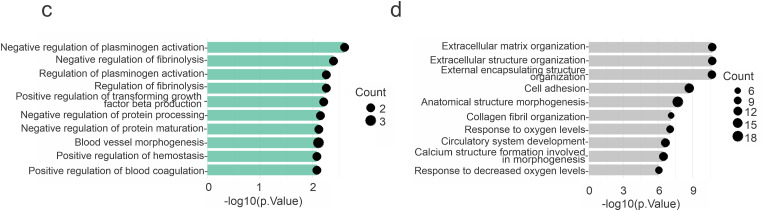
Analysis of differentially expressed proteins (DEPs) in breast cancer patients (BCPs)-conditioned media (CM) and healthy volunteer (HV)-CM. (**a**) Venn diagram illustrating the number of DEPs for each CM group. (**b**) Volcano plot showing statistical significance of DEPs (adjusted *p*-value) vs. magnitude of expression change (log2 fold change); numbers indicate the upregulated proteins in BCPs-CM (DEPs = 7) and HV-CM (DEPs = 34). (**c**) Gene Ontology (GO) biological process enrichment analysis of DEPs secreted by BCPs-MSCs. The top 10 enriched biological processes are shown. (**d**) GO biological process enrichment analysis of DEPs secreted by HV-MSCs. The top 10 enriched biological processes are shown. Bubble sizes correspond to the number of proteins associated with each process. All analyses were conducted using DAVID Bioinformatics Resources (v2023q4). DAVID: Database for Annotation, Visualization, and Integrated Discovery.

In this study, a total of 141 proteins were identified (Fig. 8a,b), of which 7 were differentially expressed in the BCPs group and 34 in the HVs group (Tables 2 and 3). The 100 proteins commonly expressed in both groups are listed in Supplementary Table S1.

**Table 2:** DEPs: BCPs-CM.

Protein Name	Accession	Mean	−Log (*p*-Value)	Difference
CCN family member 2	P29279	25.65482	3.148877	−0.972930
Thrombospondin-1	P07996	30.60858	1.836893	−1.396545
Keratin, type II cytoskeletal 74	F8W1S1	30.70347	1.790534	−1.324390
Tubulin alpha-3D chain	P0DPH8	26.81367	1.576747	−0.866872
Hemoglobin subunit alpha	P69905	30.37256	1.540205	−0.547122
Gc-globulin	D6RF35	29.81652	1.459337	−1.863735
Alpha-2-antiplasmin	P08697	28.02006	1.304293	−1.557733

Note: Proteins are ranked by increasing fold change in expression. Accession: UniProt identifier. Mean: average of the normalized counts taken over all samples. Difference: fold change in protein expression. DEPs: Differentially expressed proteins; BCPs-CM: Breast cancer patients-pooled conditioned media.

**Table 3:** DEPs: HVs-CM.

Protein Name	Accession	Mean	−Log (*p*-Value)	Difference
Biglycan	P21810	30.83386	5.495142	2.288571
Hyaluronan and proteoglycan link protein 1	P10915	28.55103	4.416551	2.364335
SPARC	P09486	33.31896	3.033892	2.196668
Collagen alpha-1(I) chain	P02452	31.43454	2.981144	1.982475
Immunoglobulin superfamily leucine-rich repeat protein	O14498	26.37549	2.980413	2.589291
Endosialin	Q9HCU0	23.94062	2.695629	2.613647
Nucleobindin-1	Q02818	24.51601	2.543104	1.612327
Collagen alpha-2(I) chain	P08123	31.46207	2.504757	1.641663
Sulfhydryl oxidase 1	O00391	28.83027	2.444776	1.880081
Collagen alpha-1(VI) chain	P12109	31.80457	2.435391	1.706615
Insulin-like growth factor-binding protein 7	Q16270	31.04168	2.434321	1.341565
Calumenin	O43852	27.05615	2.389051	2.402885
Periostin	B1ALD9	30.84445	2.347788	2.105738
Protein disulfide-isomerase	A0A7P0TA71	26.42480	2.074880	0.981511
Cystatin-C	P01034	28.10000	2.053037	1.594201
Fibronectin	P02751	32.27891	2.032473	1.334974
Metalloproteinase inhibitor 2	P16035	27.48701	2.031007	1.774174
Coiled-coil domain-containing protein 80	Q76M96	26.07931	2.006941	1.732424
Collagen alpha-1(XII) chain	Q99715	26.66183	1.869145	1.700677
Collagen alpha-2(VI) chain	P12110	27.34088	1.829460	1.934874
Calsyntenin-1	O94985	27.76339	1.714080	0.931322
Alpha-actinin-1	A0A7I2V4Y4	27.29588	1.618984	0.724749
Inhibin beta A chain	P08476	28.12416	1.607380	0.784331
Galectin-3-binding protein	Q08380	28.73935	1.591038	1.999163
Transforming growth factor-beta-induced protein	Q15582	31.07112	1.537662	0.672927
Basement membrane-specific proteoglycan core protein	P98160	27.38769	1.532514	2.307629
Beta tropomyosin isoform	A7XZE4	27.20738	1.529357	1.560976
Procollagen-lysine,2-oxoglutarate 5-dioxygenase 1	Q02809	24.91555	1.526235	2.785484
Lysyl oxidase homolog 2	Q9Y4K0	23.84312	1.461128	1.685429
Plasminogen activator inhibitor 1	P05121	31.05232	1.449524	0.902847
Cathepsin Z	A0A7P0T8I6	25.98795	1.433076	0.702921
Complement C1s subcomponent	P09871	27.67334	1.412462	2.225858
72 kDa type IV collagenase	P08253	29.20730	1.354269	2.136936
Fibrillin-1	P35555	26.30720	1.326463	1.099941

Note: Proteins are ranked by increasing fold change in expression. Accession: UniProt identifier. Mean: average of the normalized counts taken over all samples. Difference: fold change in protein expression. DEPs: Differentially expressed proteins; BCPs-CM: Breast cancer patients-pooled conditioned media; SPARC: Secreted Protein Acidic and Rich in Cysteine.

Interestingly, among the differentially secreted proteins in BCPs, cellular communication network factor 2 (CCN2), thrombospondin-1 (THBS1), α-2-antiplasmin (SERPINF2) and type II cytoskeletal keratin 74 (KRT74) stood out due to their known roles in MSC dysfunction and oxidative stress responses [20,44,45].

The top 10 significantly enriched terms (GO biological process) for BCPs and HVs are shown in Fig. 8c,d, respectively. In BCPs, additional terms, reactive oxygen species metabolic process and positive regulation of MAPK cascade, were identified, which is of particular interest due to its link to the oxidative stress previously reported in patient-derived MSCs. In HVs, additional terms such as direct ossification, intramembranous ossification, ossification, and bone trabecula formation were identified, which are noteworthy for their association with healthy MSCs and bone formation. Complete lists of enriched terms are provided in Supplementary Tables S2 and S3.

## 4 Discussion

Dysfunctional MSCs promote the metastatic cascade by creating the BM/ bone PMN [16,21,46,47].

Investigating the mechanisms underlying PMN formation and their role in tumor invasion and outgrowth remains an active area of research [22,48]. However, the impact of increased oxidative stress on MSCs in advanced BCPs, and how it alters their physiological functions remains poorly understood. Here, we provide compelling evidence of such alterations in MSCs from untreated advanced BCPs. It has been reported that soluble factors such as CCL2, acting through its receptor CCR2, reinforce senescence in MSCs by upregulating p53 and p21 protein levels via ROS-mediated or p38-MAPK signaling pathways [42]. Notably, p53 activation in senescent cells further promotes CCL2 secretion, establishing a feed-forward loop that sustains the senescent phenotype. Consistent with this mechanism, we previously showed that in advanced BCPs (clinic-pathological stage III-B) the BM microenvironment shifts toward a pro-osteoclastogenic and inflammatory state, characterized by elevated CCL-2 levels even before detectable bone or BM metastasis [20]. In line with these findings, we now report an increased proportion of patient-derived MSCs expressing CCR2, indicative of a microenvironment prone to oxidative stress and ROS generation. Given that cellular senescence can propagate in a paracrine manner, a relatively small CCR2^+^ MSCs subpopulation may be sufficient to initiate a feed-forward loop in which senescent cells induce senescence in neighboring MSCs [49], leading to a progressive expansion of the senescent MSCs pool.

Consistently, MSCs from BCPs exhibited elevated expression of oxidative stress response genes, including HSP90 and Nrf2, which are induced by oxidative stress and play a key role in protein protection and repair, as well as in the regulation of antioxidant genes such as SOD1, SOD2, CAT, and GPx-1. Although activation of these antioxidant pathways helps counteract ROS and preserve cellular homeostasis [50–52], sustained oxidative stress ultimately leads to cumulative biomolecular damage [26,53]. Consistent with this, we observed significantly increased DNA damage in MSCs from BCPs compared with those from HVs, as assessed by the alkaline comet assay. This sensitive technique detects DNA strand breaks and alkali-labile sites associated with ROS-induced genomic damage [33,54]. The extent of DNA damage critically influences cell fate decisions, as both DNA repair and apoptotic pathways are initiated by cell cycle arrest, in agreement with our previous observations of impaired cell cycle progression in these MSCs [16].

Despite the presence of DNA repair mechanisms, cells do not always successfully reverse damage, and some lesions may remain unrepaired or be misrepaired. Such residual lesions can trigger apoptosis; however, when apoptotic pathways are compromised, the risk of chromosomal instability increases. This instability includes numerical chromosomal alterations and structural CAs, such as the TAS predominantly observed in MSCs from BCPs. The formation of these structural CAs requires breakage of chromatids at chromosome ends followed by end-to-end fusion [55].

In this context, telomeres are especially vulnerable to oxidative damage due to their high guanine content [56,57]. Under conditions of increased oxidative stress, as observed in BCPs, single-strand DNA breaks preferentially accumulate in telomeres. This leads to instability in this chromosomal region [58], which could explain why TAS represent the most frequent CA observed in MSCs from these patients.

As a consequence of defective DNA repair under oxidative stress, MSCs from BCPs also exhibit NBs and MN. These nucleus-like structures arise from lagging chromosomes or chromosomal fragments that fail to be reincorporated into the primary nucleus following cell division [59]. Both NBs and MN are widely recognized as biomarkers of genomic instability and are frequently associated with impaired DNA repair mechanisms [60]. Excessive ROS can damage DNA and cell-cycle–associated proteins, thereby compromising genetic stability and proper chromosomal segregation [33,61], which likely explains the increased frequency of NBs and MN observed in MSCs from BCPs. Notably, Sharma et al. demonstrated that elevated NB and MN frequencies impair MSC proliferation and alter cell cycle progression through differential regulation of cell cycle checkpoint genes associated with cell cycle arrest [62]. These findings are consistent with our previous observations showing reduced self-renewal capacity, prolonged G0/G1 phase, and shortened S phase in MSCs from this patient group [16].

Similarly, lipid oxidation was increased, as evidenced by elevated levels of 4-HNE.This reactive lipid peroxidation product inhibits DNA, RNA, and protein synthesis and exerts both genotoxic and cytotoxic effects [63,64]. Beyond serving as a biomarker of lipid peroxidation, 4-HNE is highly reactive and forms adducts with DNA and proteins, thereby constituting an additional indicator of ROS-induced molecular damage [65]. Notably, it has been reported that an increase in 4-HNE is dose-dependently linked to the increase in Nrf2 expression, which leads to an increase in the expression of antioxidant genes, as demonstrated in BCP samples. Moreover, *in vitro* experiments showed that 4-HNE induced significantly elevated levels of CAs and MN, in certain cell types [64]. This last observation partially explains the increase in the number of CAs and MN observed in MSCs from BCPs, as it links them to the elevated levels of 4-HNE present in these stromal cells.

Notably, protein carbonylation, the primary product of ROS-mediated oxidation reactions in proteins, has been found to be increased in protein isolates from the lysates of BCPs-MSCs, as well as in their CM. This observation is consistent with oxidative stress affecting multiple components of the proteome in these cells. Krisko Radman described that protein carbonylation serves as an indicator of diminished functionality of a damaged proteome [66]. Particularly, telomerase, one of the least abundant cellular proteins, is among the human proteins most susceptible to carbonylation [67], which could help explain the reduced telomerase activity previously reported in MSCs from these patients [16]. Moreover, telomere shortening and genomic instability can be considered secondary consequences of widespread proteome damage [68,69].

Our findings reveal substantial damage to DNA, proteins, and lipids in BCPs-MSCs, alterations that will inevitably impact the relationship between these cells and their surrounding environment [70]. Accordingly, characterization of the MSC secretome is essential to understand how BCP-derived MSCs contribute to PMN formation within the BM/bone microenvironment, as well as how MSCs from HVs regulate normal BM homeostasis.

Among the differentially expressed proteins identified in the secretome of BCPs-MSCs, the GO analysis highlighted biological processes relevant to the pathophysiological context of the PMN. These included pathways related to cellular signaling (positive regulation of transforming growth factor beta production and positive regulation of MAPK cascade), oxidative stress (reactive oxygen species metabolic process), and protein homeostasis (negative regulation of protein processing and negative regulation of protein maturation). Collectively, these processes reinforce the notion that BCPs-MSCs actively contribute to the remodeling of the BM microenvironment into PMN. These associations were primarily driven by the expression of CCN2, THBS1, SERPINF2, KRT74, and the vitamin D-binding protein (GC).

Regarding CCN2, the members of the CCN family are proteins present in the extracellular matrix. Overexpression of CCN2 is closely related to cell adhesion, proliferation, and migration, directly contributing to tumor development and metastasis in various types of cancer. Specifically in breast cancer, CCN2 can regulate the expression of matrix metalloproteinase-1, transforming growth factor-beta (TGF-β), N-cadherin, Snail, β-catenin, and bone morphogenetic protein (BMP), promoting metastasis and angiogenesis [71,72]. TGF-β signaling is particularly relevant, as it drives epithelial mesenchymal transition and promotes tumor cell invasion and metastasis in advanced stages of breast cancer. Under hypoxic conditions, TGF-β enhances the hypoxia-inducible factor-1 signaling pathway within the bone microenvironment, stimulating osteoclast formation and activity while inhibiting the osteogenic differentiation of MSCs [73–77]. Notably, TGF-β1 promotes senescence of BM-MSCs through elevated mitochondrial ROS production [78]. In the BM, CCN2 is mainly expressed by MSCs, where it supports early osteogenic differentiation but inhibits Wnt3a- and BMP-induced differentiation when persistently expressed. Moreover, by interacting with both receptor-receptor activator of nuclear factor kappa-B ligand osteoprotegerin, CCN2 enhances receptor activator of nuclear factor kappa-B ligand signaling, thereby promoting osteoclast formation and activity [72,79,80].

In summary, CCN2 expression may partly account for the impaired osteogenic differentiation of BCP-derived MSCs and the enhanced osteoclastic activity previously reported in the PMN of these patients, as well as for the promotion of MSC senescence through mitochondrial ROS production [20,21].

With regard to THBS1, functions as an an inhibitor of stem cell self-renewal. Acting through its cell surface receptor CD47, THBS1 suppresses the expression of important self-renewal transcription factors, including OCT4, SOX2, KLF4, and MYC in non-malignant cells [45]. This mechanism could underlie the decreased colony-forming ability and the reduced expression of stemness-associated genes observed in MSCs from these patients [16]. In addition, THBS1 may contribute to the PMN formation by disrupting the endothelial intercellular junctions integrity, a process known to facilitate breast cancer metastasis [81].

Both THBS1 and SERPINF2 have been reported as plasma biomarkers of breast disease [82,83]. Our findings suggest that BM-derived MSCs from these patients could represent a potential source of these elevated factors.

In addition to these proteins, keratins, including KRT74, are epithelial markers associated with cancer progression, diagnosis, and treatment, and their expression is often elevated in cancer or oxidative stress conditions [44], which is relevant given the increased oxidative stress observed in BCPs-MSCs [16].

Finally, GC, a vitamin D-related protein involved in bone metabolism, can bind C5a, extending its half-life and activity. C5a promotes osteoclast activation and, in the presence of IL-1β, induces IL-6 and IL-8 expression [84,85], which may contribute to the pro-inflammatory PMN and increased osteoclastogenesis observed in these patients [16,20].

The remaining two proteins identified in the BCP-derived MSC secretome lack well-defined roles in cancer or BM biology and were therefore not discussed further.

The secretome of MSCs derived from HVs was was enriched in proteins whose GO annotations highlighted biological processes fundamental to maintaining a healthy tissue microenvironment, including extracellular matrix organization, extracellular structure organization, cell adhesion, regulation of cell population proliferation, regulation of cell migration, and ossification.

Among the identified proteins are collagen alpha-2(I) chain (COL1A2), secreted protein acidic and rich in cysteine (SPARC), and quiescin sulfhydryl oxidase 1 (QSOX1) were particularly notable. We focused on these proteins given their direct relevance to bone homeostasis, whereas the remaining proteins, although important for general tissue maintenance, have less clearly defined roles in these processes.

Regarding COL1A2, this protein supports type I collagen synthesis, fibroblast proliferation, elastin production, and wound healing, thereby playing a key role in bone formation [86]. SPARC regulates bone remodeling, inhibits breast cancer cell migration, and suppresses osteoclast activation; notably, reduced stromal SPARC levels have been associated with bone metastasis [87,88]. QSOX1 is essential for laminin incorporation, cell adhesion, and migration, stabilizes tissue inhibitors of metalloproteinases (TIMPs) to inhibit matrix metalloproteinase activity, and protects against oxidative stress. Importantly, higher QSOX1 expression in BCPs has been correlated with improved patient survival [89–91]. This profile contrasts markedly with the secretome of MSCs derived from BCPs, in which the microenvironment is remodeled to support tumor progression. These findings suggest that alterations in BCP-derived MSCs drive a shift in the secreted protein profile, leading to dysfunction in key processes such as bone formation and angiogenesis—both essential for maintaining BM/bone homeostasis. Disruption of these functions appears sufficient to promote PMN formation, as observed in this patient group. Nevertheless, future quantitative and functional assays will be required to validate and further characterize the secretome-associated changes in BCP-derived MSCs. Taken together, the alterations observed in the secretome of BCP-derived MSCs reflect a broader dysfunctional phenotype characterized by cellular senescence, metabolic reprogramming, and molecular changes that favor PMN establishment [16,20,21]. In this context, senescent MSCs from these patients exhibit the metabolic and molecular features required to support PMN formation within the BM/bone microenvironment. Because disease progression largely depends on PMN characteristics—the so-called “fertile soil”—which are in turn shaped by MSCs, therapeutic strategies aimed at normalizing MSC function are of particular interest. Restoration of this altered MSC phenotype could delay BM/bone metastasis and improve patient quality of life. However, reversing the damage caused by chronic oxidative stress in patients at this stage of the disease (IIIB) remains challenging [92]. Future studies should explore antioxidants—such as resveratrol, ascorbic acid, and idebenone—or their combinations, as well as their potential use alongside additional interventions, including anti-CCL2 or anti-CCR2 therapies [93,94]. In addition, senolytic approaches, such as treatment with quercetin, which has been shown to effectively eliminate senescent MSCs, may represent a promising strategy to purify the MSC pool by selectively removing senescent cells [95–98].

Nevertheless, the temporal interval between primary tumor diagnosis and metastatic dissemination highlights the potential of early therapeutic strategies aimed at preserving BM-MSC health to prevent senescence-driven niche formation.

## 5 Limitations

1. Small sample size.

This study is limited by the relatively small number of patient-derived MSC samples (n = 10), which may affect statistical power and reflects the strict inclusion and exclusion criteria applied for patient selection.

2. Variable sample numbers across assays.

The number of samples included in each experimental assay varied due to the intrinsically low self-renewal capacity of MSCs obtained from untreated advanced BCPs, which limited cell expansion and the availability of sufficient material for all downstream analyses.

Despite these constraints, the converging evidence obtained from multiple complementary analyses strengthens the reliability of the observed trends.

## 6 Conclusion

Our findings define a distinct oxidative stress signature in BM–derived MSCs from untreated advanced BCPs, characterized by the accumulation of DNA damage, genomic instability, increased protein carbonylation, and enhanced lipid peroxidation. These oxidative stress–associated alterations are accompanied by dysregulated expression of oxidative stress response genes and marked changes in the MSCs secretome compared with HVs. Collectively, this molecular footprint provides mechanistic insight into PMN formation and may support the development of prognostic biomarkers and personalized therapeutic strategies aimed at delaying or preventing bone metastasis.

## Supplementary Materials



## Data Availability

The datasets generated or analyzed during the current study are available from the corresponding authors on reasonable request.

## Supplementary Materials

The supplementary material is available online at https://www.techscience.com/doi/10.32604/or.2026.074321/s1.

## Nomenclature

α-MEM: α-minimal essential medium
ACs: Abnormal cells
Abs: Antibodies
AE1–AE3: Cytokeratins
AF: Acentric fragments
BM: Bone Marrow
BMP: Bone morphogenetic proteins
BCPs: Breast Cancer Patients
BSA: Bovine serum albumin
CAs: Chromosomal aberrations
CAT: Catalase
CB: Chromatid breaks
CCL-2: C-C motif chemokine 2
CCR2: C-C motif chemokine receptor 2
cDNA: Complementary DNA
CFU-F: Fibroblastic Colony-Forming Unit
CM: Conditioned Media
DIC: Dicentric chromosomes
DNA: Deoxyribonucleic Acid
DNPH: 2,4-dinitrophenylhydrazine
DTT: Dithiothreitol
EDTA: Ethylene diamine tetraacetic acid
EMA: Epithelial membrane antigen
FBS: Fetal Bovine Serum
GAPDH: Glyceraldehyde-3-phosphate dehydrogenase
GO: Gene Ontology
GPx1: Glutathione Peroxidase 1
HRP: Horseradish Peroxidase
HSP90: Heat Shock Protein 90
HUC-MSCs: Human Umbilical Cord Mesenchymal Stem Cells
HVs: Healthy volunteers
TERT: Telomerase Reverse Transcriptase
IL-6: Interleukin 6
LFQ: Label-Free Quantification
LMP: Low-melting-point agarose
MN: Micronuclei
MNCs: Mononuclear cells
MSCs: Mesenchymal Stem/ Stromal Cells
NBs: Nuclear blebs
NMP: Normal-melting-point agarose
Nrf2: Nuclear Factor Erythroid 2–Related Factor 2
OCT4: Octamer-binding transcription factor 4
PBS: Phosphate-Buffered Saline
PFA: Paraformaldehyde
PMN: Pre-Metastatic Niche
RC: Ring chromosomes
RNA: Ribonucleic acid
ROS: Reactive Oxygen Species
RT-PCR: Reverse Transcription Polymerase Chain Reaction
SASP: Senescence-Associated Secretory Phenotype
SDS-PAGE: Sodium dodecyl sulfate-polyacrylamide
SE: Standard Error
SOD1: Superoxide Dismutase 1
SOD2: Superoxide Dismutase 2
SOX2: Sex determining region Y-box 2
TAS: Telomeric associations
TBARS: 2-thiobarbituric acid-reactive substances
TBS: Tris-Buffered Saline
T-20: Tween 20
TGF-β: Transforming growth factor beta
TNF-α: Tumor Necrosis Factor Alpha
VEGF: Vascular Endothelial Growth Factor
4-HNE: 4-Hydroxynonenal
