# Supplementary material for: Oxidative Stress Footprints in Bone Marrow Mesenchymal Stem Cells from Untreated Advanced Breast Cancer
Source: Oncol Res. 2026 Mar 23;34(4):20. doi: 10.32604/or.2026.074321 (PMC13040344; doi:10.32604/or.2026.074321)
Supplement: Supplementary file 1 [file OncolRes-34-74321-s001.docx]

| **Table S1:** Non-DEPs between HVs-CM and BCPs-CM | | | | |
| --- | --- | --- | --- | --- |
| **Protein name** | **Accession** | **Mean** | **­Log (p-value)** | **Difference** |
| Albumin | P02768 | 38,80974 | 0,533483 | -0,090469 |
| Keratin, type II cytoskeletal 1 | P04264 | 36,94108 | 0,184582 | 0,094969 |
| Keratin, type I cytoskeletal 10 | A0A1B0GVI3 | 36,01796 | 0,061869 | 0,029282 |
| Keratin, type II cytoskeletal 2 epidermal | P35908 | 35,65384 | 0,110197 | 0,064573 |
| Keratin, type I cytoskeletal 9 | P35527 | 35,10745 | 0,756929 | -0,266549 |
| Lactotransferrin | P02788 | 33,87601 | 0,110295 | 0,129927 |
| Keratin, type I cytoskeletal 14 | P02533 | 33,28204 | 0,315980 | 0,164272 |
| Keratin, type II cytoskeletal 5 | P13647 | 32,86334 | 0,146856 | 0,087018 |
| Actin, cytoplasmic 1 | P60709 | 32,59562 | 0,103709 | 0,035573 |
| Glia-derived nexin | P07093 | 31,58211 | 0,530347 | 0,369754 |
| Lumican | P51884 | 30,15888 | 1,108314 | 2,868247 |
| Keratin, type II cytoskeletal 6A | P02538 | 31,70847 | 0,317916 | 0,349992 |
| Keratin, type I cytoskeletal 13 | P13646 | 31,64447 | 0,187021 | -0,442366 |
| Inter-alpha-trypsin inhibitor heavy chain H2 | P19823 | 31,05160 | 0,259478 | -0,126191 |
| Testis-specific Y-encoded-like protein 2 | Q9H2G4 | 32,32866 | 0,805202 | -2,222813 |
| Metalloproteinase inhibitor 1 | P01033 | 30,66374 | 0,123045 | 0,087980 |
| Dermcidin | P81605 | 30,52635 | 0,430225 | 0,109296 |
| Follistatin-related protein 1 | Q12841 | 29,35996 | 1,007614 | 2,098656 |
| Pigment epithelium-derived factor | P36955 | 30,07983 | 0,542764 | 0,430979 |
| Annexin | H0YMW4 | 30,19342 | 0,174520 | -0,216153 |
| Alpha-2-macroglobulin | P01023 | 29,64484 | 0,099637 | 0,073759 |
| Desmoplakin | P15924 | 29,79807 | 0,671261 | 0,231524 |
| Alpha-fetoprotein | J3KMX3 | 29,96806 | 0,029905 | 0,079058 |
| Complement C3 | P01024 | 29,44232 | 0,119060 | -0,118802 |
| Prolactin-inducible protein | P12273 | 29,47401 | 0,311142 | -0,392537 |
| Cathepsin D | C9JH19 | 29,08338 | 1,298723 | 0,565962 |
| Keratin, type I cytoskeletal 16 | P08779 | 29,44487 | 0,087573 | 0,127075 |
| Pentraxin-related protein PTX3 | P26022 | 28,87560 | 0,266881 | 0,615465 |
| Alpha-enolase | P06733 | 28,93228 | 0,975986 | 0,566453 |
| EGF-like I-like domain-containing protein 3 | O43854 | 29,49507 | 0,724372 | -0,886371 |
| Hornerin | Q86YZ3 | 28,70017 | 0,555279 | 0,704813 |
| Serpin H1 | P50454 | 27,72482 | 1,161237 | 1,941720 |
| Insulin-like growth factor-binding protein 3 | P17936 | 28,94652 | 0,569118 | 0,162121 |
| Keratin, type I cytoskeletal 17 | Q04695 | 28,76945 | 0,234909 | 0,587279 |
| Alpha-1B-glycoprotein | P04217 | 28,21732 | 0,348741 | 1,244715 |
| Thrombospondin-2 | A0A3B3ITK0 | 28,25260 | 0,783374 | 1,258465 |
| Fibulin-1 | P23142 | 28,82406 | 0,043015 | -0,054153 |
| Serpin A12 | Q8IW75 | 27,67740 | 0,455339 | 1,402201 |
| L-lactate dehydrogenase A chain | P00338 | 28,51054 | 0,432164 | 0,329882 |
| Keratin, type II cytoskeletal 6B | P04259 | 28,81796 | 0,004873 | -0,002599 |
| Protein S100-A8 | P05109 | 28,45530 | 0,013671 | -0,017591 |
| Semenogelin-1 | P04279 | 29,46631 | 0,460667 | -1,314623 |
| Keratin, type II cytoskeletal 78 | Q8N1N4 | 28,68014 | 0,091310 | -0,111960 |
| Collagen alpha-1(V) chain | P20908 | 28,53340 | 0,984114 | 1,960135 |
| Carboxypeptidase A4 | Q9UI42 | 27,86812 | 0,820000 | 1,058832 |
| Heat shock cognate 71 kDa protein | P11142 | 27,34051 | 0,413321 | 0,853558 |
| Gelsolin | P06396 | 27,71740 | 0,339074 | 0,567390 |
| Inter-alpha-trypsin inhibitor heavy chain H3 | Q06033 | 29,02838 | 0,873457 | -0,619414 |
| Vimentin | P08670 | 28,40060 | 0,038818 | 0,073936 |
| Protein S100-A9 | P06702 | 27,99920 | 0,534697 | 0,713338 |
| Hemoglobin subunit beta | P68871 | 28,22894 | 0,734490 | -0,727581 |
| Desmocollin-1 | Q08554 | 27,86686 | 0,002680 | -0,002341 |
| Glyceraldehyde-3-phosphate dehydrogenase | P04406 | 28,00923 | 0,674871 | -0,337564 |
| Profilin-1 | P07737 | 27,05048 | 0,422211 | 0,452162 |
| Serpin B12 | Q96P63 | 27,77396 | 0,171387 | 0,229726 |
| Pyruvate kinase PKM | P14618 | 27,52316 | 0,886754 | 0,779388 |
| Trypsin-3 | P35030 | 28,05886 | 0,031322 | 0,038354 |
| Immunoglobulin heavy constant alpha 1 | A0A286YEY1 | 27,83306 | 0,354111 | 0,318553 |
| TGc domain-containing protein | A0A494C0J7 | 27,85018 | 1,160787 | -0,643680 |
| Junction plakoglobin | P14923 | 28,27964 | 0,067885 | 0,132842 |
| Lactadherin | Q08431 | 27,29469 | 0,261923 | -0,611531 |
| Semenogelin-2 | Q02383 | 28,13800 | 0,358702 | -1,132639 |
| Calmodulin-like protein 5 | Q9NZT1 | 26,82837 | 0,750868 | 0,544868 |
| Fructose-bisphosphate aldolase | J3KPS3 | 27,20651 | 0,176660 | 0,120961 |
| Desmoglein-1 | Q02413 | 27,80642 | 0,079279 | 0,080158 |
| Secretoglobin family 1D member 2 | O95969 | 28,03429 | 0,845036 | -0,548036 |
| Keratin, type II cytoskeletal 1b | Q7Z794 | 27,22612 | 0,129788 | 0,054214 |
| Thioredoxin | P10599 | 26,94043 | 0,083976 | 0,111056 |
| Protein S100-A7 | P31151 | 26,83165 | 0,536613 | -0,638838 |
| Ubiquitin-40S ribosomal protein S27a | P62979 | 28,25214 | 0,322035 | -0,393855 |
| Peroxiredoxin-1 | Q06830 | 26,97254 | 0,152481 | -0,079116 |
| Zinc-alpha-2-glycoprotein | P25311 | 26,71509 | 0,162666 | 0,329580 |
| Histone H2A | A0A0U1RRH7 | 26,94618 | 0,397472 | -0,282309 |
| Arginase-1 | P05089 | 28,41373 | 0,252015 | -0,473253 |
| Adenosylhomocysteinase | P23526 | 26,21563 | 0,004748 | -0,004750 |
| Protein-lysine 6-oxidase | P28300 | 26,63457 | 0,754614 | -0,423050 |
| Alpha-2-HS-glycoprotein | C9JV77 | 26,30162 | 1,312941 | 0,890907 |
| Proteasome subunit beta type-6 | P28072 | 25,77367 | 0,834878 | 0,916971 |
| Triosephosphate isomerase | P60174 | 26,19025 | 0,922397 | 0,426704 |
| Immunoglobulin heavy constant gamma 1 | A0A0A0MS08 | 26,25318 | 0,004436 | 0,002428 |
| Elongation factor 2 | P13639 | 25,62253 | 0,431240 | 1,251452 |
| Complement component C9 | P02748 | 26,17181 | 0,763262 | -0,547800 |
| Cartilage oligomeric matrix protein | P49747 | 26,26276 | 0,214532 | -0,181067 |
| Cilia- and flagella-associated protein 100 | Q494V2 | 26,92530 | 1,157752 | -1,667334 |
| EEF1A lysine methyltransferase 1 | Q8WVE0 | 32,95772 | 0,512524 | -3,065777 |
| Zymogen granule protein 16 homolog B | Q96DA0 | 27,23472 | 0,807196 | -0,777292 |
| Vasorin | Q6EMK4 | 25,25310 | 1,137512 | 1,001442 |
| Heat shock 27 kDa protein | A0A6Q8PGK1 | 26,88214 | 0,398557 | -0,553926 |
| Immunoglobulin lambda constant 2 | P0DOY2 | 26,24764 | 0,521485 | -0,608407 |
| Hepatocyte growth factor activator | D6RAR4 | 25,61039 | 0,226581 | -0,158740 |
| Complement component C7 | P10643 | 25,71214 | 0,100435 | -0,122075 |
| Insulin-like growth factor-binding protein 4 | P22692 | 26,75216 | 0,270757 | -0,689268 |
| Annexin A5 | P08758 | 26,04895 | 0,533321 | -0,578490 |
| Keratin, type II cytoskeletal 80 | Q6KB66 | 24,60577 | 0,564599 | 1,663130 |
| Peptidyl-prolyl cis-trans isomerase B | P23284 | 25,28796 | 0,388266 | -0,595111 |
| Mammaglobin-A | Q6NX70 | 25,72659 | 0,616099 | -0,578475 |
| Prelamin-A/C | A0A6Q8PFJ0 | 24,64673 | 0,677928 | 2,106499 |
| Endoplasmic reticulum chaperone BiP | P11021 | 25,51156 | 0,082520 | 0,112272 |
| Filaggrin-2 | Q5D862 | 25,40717 | 0,230474 | 0,218686 |
| Tubulin alpha chain | F5H5D3 | 24,26907 | 0,383707 | -0,854816 |

Note: Non-DEPs: Non-Differential expressed proteins. BCPs-CM: Breast cancer patients-conditioned media. Proteins are ranked by increasing fold change in expression. Accession: UniProt identifier. Mean: average of the normalized counts taken over all samples. Difference: fold change in protein expression.

| **Table S2:** Biological process enrichment analysis of DEPs in BCPs-CM | | | | | | | |
| --- | --- | --- | --- | --- | --- | --- | --- |
| **Term** | **Count** | **List Total** | **Pop Hits** | **Pop Total** | **P-Value** | **Fold Enrichment** | **FDR** |
| **Negative Regulation of Plasminogen Activation** | 2 | 7 | 8 | 19416 | 0,00247 | 693,43 | 0,415 |
| **Negative Regulation of Fibrinolysis** | 2 | 7 | 13 | 19416 | 0,00401 | 426,73 | 0,415 |
| **Regulation of Plasminogen Activation** | 2 | 7 | 18 | 19416 | 0,00555 | 308,19 | 0,415 |
| **Regulation of Fibrinolysis** | 2 | 7 | 18 | 19416 | 0,00555 | 308,19 | 0,415 |
| **Positive Regulation of Transforming Growth Factor Beta Production** | 2 | 7 | 20 | 19416 | 0,00617 | 277,37 | 0,415 |
| **Negative Regulation of Protein Processing** | 2 | 7 | 23 | 19416 | 0,00709 | 241,19 | 0,415 |
| **Negative Regulation of Protein Maturation** | 2 | 7 | 25 | 19416 | 0,0077 | 221,90 | 0,415 |
| **Blood Vessel Morphogenesis** | 3 | 7 | 457 | 19416 | 0,00779 | 18,21 | 0,415 |
| **Positive Regulation of Hemostasis** | 2 | 7 | 27 | 19416 | 0,00832 | 205,46 | 0,415 |
| **Positive Regulation of Blood Coagulation** | 2 | 7 | 27 | 19416 | 0,00832 | 205,46 | 0,415 |
| Positive Regulation of Mapk Cascade | 3 | 7 | 484 | 19416 | 0,0087 | 17,19 | 0,415 |
| Positive Regulation of Coagulation | 2 | 7 | 31 | 19416 | 0,00954 | 178,95 | 0,417 |
| Regulation of Transforming Growth Factor Beta Production | 2 | 7 | 34 | 19416 | 0,0105 | 163,16 | 0,422 |
| Blood Vessel Development | 3 | 7 | 555 | 19416 | 0,0113 | 14,99 | 0,424 |
| Vasculature Development | 3 | 7 | 577 | 19416 | 0,0122 | 14,42 | 0,427 |
| Positive Regulation of Stress Fiber Assembly | 2 | 7 | 53 | 19416 | 0,0163 | 104,67 | 0,475 |
| Regulation of Mapk Cascade | 3 | 7 | 690 | 19416 | 0,0172 | 12,06 | 0,475 |
| Regulation of Protein Processing | 2 | 7 | 60 | 19416 | 0,0184 | 92,46 | 0,475 |
| Tube Morphogenesis | 3 | 7 | 717 | 19416 | 0,0185 | 11,61 | 0,475 |
| Positive Regulation of Actin Filament Bundle Assembly | 2 | 7 | 63 | 19416 | 0,0193 | 88,05 | 0,475 |
| Positive Regulation of Wound Healing | 2 | 7 | 66 | 19416 | 0,0202 | 84,05 | 0,475 |
| Regulation of Protein Maturation | 2 | 7 | 66 | 19416 | 0,0202 | 84,05 | 0,475 |
| Regulation of Blood Coagulation | 2 | 7 | 69 | 19416 | 0,0211 | 80,40 | 0,475 |
| Regulation of Hemostasis | 2 | 7 | 71 | 19416 | 0,0217 | 78,13 | 0,475 |
| Regulation of Coagulation | 2 | 7 | 77 | 19416 | 0,0236 | 72,04 | 0,494 |
| Positive Regulation of Response to Wounding | 2 | 7 | 82 | 19416 | 0,0251 | 67,65 | 0,505 |
| Regulation of Stress Fiber Assembly | 2 | 7 | 99 | 19416 | 0,0302 | 56,03 | 0,537 |
| Positive Regulation of Jnk Cascade | 2 | 7 | 99 | 19416 | 0,0302 | 56,03 | 0,537 |
| Positive Regulation of Smooth Muscle Cell Proliferation | 2 | 7 | 101 | 19416 | 0,0308 | 54,93 | 0,537 |
| Tube Development | 3 | 7 | 947 | 19416 | 0,0313 | 8,79 | 0,537 |
| Circulatory System Development | 3 | 7 | 980 | 19416 | 0,0333 | 8,49 | 0,537 |
| Regulation of Actomyosin Structure Organization | 2 | 7 | 110 | 19416 | 0,0335 | 50,43 | 0,537 |
| Regulation of Actin Filament Bundle Assembly | 2 | 7 | 111 | 19416 | 0,0338 | 49,98 | 0,537 |
| **Reactive Oxygen Species Metabolic Process** | 2 | 7 | 116 | 19416 | 0,0353 | 47,82 | 0,544 |
| Regulation of Cellular Component Biogenesis | 3 | 7 | 1070 | 19416 | 0,0392 | 7,78 | 0,587 |

Note: DEPs: Differential expressed proteins. BCPs-CM: Breast cancer patients-conditioned media. Due to the small number of proteins, these terms did not reach statistical significance after FDR correction.

| **Table S3:** Biological process enrichment analysis of DEPs in HVs-CM | | | | | | | |
| --- | --- | --- | --- | --- | --- | --- | --- |
| **Term** | **Count** | **List total** | **Pop Hits** | **Pop total** | **P-Value** | **Fold Enrichment** | **FDR** |
| **Extracellular Matrix Organization** | 11 | 33 | 286 | 19416 | 1,99E-11 | 22,63 | 8,64E-09 |
| **Extracellular Structure Organization** | 11 | 33 | 287 | 19416 | 2,06E-11 | 22,55 | 8,64E-09 |
| **External Encapsulating Structure Organization** | 11 | 33 | 288 | 19416 | 2,13E-11 | 22,47 | 8,64E-09 |
| **Cell Adhesion** | 14 | 33 | 989 | 19416 | 2,02E-09 | 8,33 | 0,000000614 |
| **Anatomical Structure Morphogenesis** | 18 | 33 | 2331 | 19416 | 2,01E-08 | 4,54 | 0,00000488 |
| **Collagen Fibril Organization** | 6 | 33 | 66 | 19416 | 0,000000073 | 53,49 | 0,0000148 |
| **Response to Oxygen Levels** | 9 | 33 | 358 | 19416 | 8,83E-08 | 14,79 | 0,0000153 |
| **Circulatory System Development** | 12 | 33 | 980 | 19416 | 0,000000248 | 7,20 | 0,0000377 |
| **Anatomical Structure Formation Involved In Morphogenesis** | 12 | 33 | 1014 | 19416 | 0,00000035 | 6,96 | 0,0000472 |
| **Response to Decreased Oxygen Levels** | 8 | 33 | 328 | 19416 | 0,000000862 | 14,35 | 0,000105 |
| **Endodermal Cell Differentiation** | 5 | 33 | 47 | 19416 | 0,00000103 | 62,59 | 0,000114 |
| Tube Development | 11 | 33 | 947 | 19416 | 0,00000176 | 6,83 | 0,000178 |
| Endoderm Formation | 5 | 33 | 57 | 19416 | 0,00000226 | 51,61 | 0,000211 |
| Blood Vessel Development | 9 | 33 | 555 | 19416 | 0,00000243 | 9,54 | 0,000211 |
| Vasculature Development | 9 | 33 | 577 | 19416 | 0,00000325 | 9,18 | 0,000263 |
| Supramolecular Fiber Organization | 9 | 33 | 633 | 19416 | 0,00000642 | 8,37 | 0,000488 |
| Response to Hypoxia | 7 | 33 | 312 | 19416 | 0,0000105 | 13,20 | 0,000721 |
| Endoderm Development | 5 | 33 | 84 | 19416 | 0,0000107 | 35,02 | 0,000721 |
| Tube Morphogenesis | 9 | 33 | 717 | 19416 | 0,0000159 | 7,39 | 0,00102 |
| Multicellular Organism Development | 19 | 33 | 4154 | 19416 | 0,0000174 | 2,69 | 0,00106 |
| Skeletal System Development | 8 | 33 | 524 | 19416 | 0,0000188 | 8,98 | 0,00109 |
| Tissue Development | 13 | 33 | 1870 | 19416 | 0,0000221 | 4,09 | 0,00122 |
| Angiogenesis | 7 | 33 | 361 | 19416 | 0,0000239 | 11,41 | 0,00126 |
| Regulation of Cell-Substrate Adhesion | 6 | 33 | 230 | 19416 | 0,0000346 | 15,35 | 0,00175 |
| Regulation of Cell Adhesion | 9 | 33 | 837 | 19416 | 0,0000482 | 6,33 | 0,00234 |
| Anatomical Structure Development | 21 | 33 | 5453 | 19416 | 0,0000506 | 2,27 | 0,00237 |
| Formation of Primary Germ Layer | 5 | 33 | 126 | 19416 | 0,0000528 | 23,35 | 0,00238 |
| Response to Abiotic Stimulus | 10 | 33 | 1179 | 19416 | 0,0000855 | 4,99 | 0,00371 |
| Blood Vessel Morphogenesis | 7 | 33 | 457 | 19416 | 0,0000887 | 9,01 | 0,00371 |
| System Development | 17 | 33 | 3758 | 19416 | 0,0000941 | 2,66 | 0,00381 |
| Animal Organ Development | 15 | 33 | 3017 | 19416 | 0,000135 | 2,93 | 0,0053 |
| Negative Regulation of Cell Adhesion | 6 | 33 | 321 | 19416 | 0,000167 | 11,00 | 0,00634 |
| Collagen Metabolic Process | 4 | 33 | 66 | 19416 | 0,000173 | 35,66 | 0,00638 |
| Animal Organ Morphogenesis | 9 | 33 | 1013 | 19416 | 0,000183 | 5,23 | 0,00654 |
| Gastrulation | 5 | 33 | 179 | 19416 | 0,000205 | 16,43 | 0,00713 |
| Developmental Process | 21 | 33 | 5992 | 19416 | 0,000217 | 2,06 | 0,00731 |
| Response to Activity | 4 | 33 | 85 | 19416 | 0,000366 | 27,69 | 0,012 |
| Embryonic Morphogenesis | 7 | 33 | 610 | 19416 | 0,000423 | 6,75 | 0,0135 |
| Positive Regulation of Cell Migration | 7 | 33 | 619 | 19416 | 0,000458 | 6,65 | 0,0143 |
| Cellular Response to Amino Acid Stimulus | 4 | 33 | 94 | 19416 | 0,000492 | 25,04 | 0,015 |
| Positive Regulation of Cell Motility | 7 | 33 | 649 | 19416 | 0,000588 | 6,35 | 0,0174 |
| Cellular Response to Acid Chemical | 4 | 33 | 103 | 19416 | 0,000643 | 22,85 | 0,0186 |
| Positive Regulation of Locomotion | 7 | 33 | 665 | 19416 | 0,000668 | 6,19 | 0,0189 |
| Negative Regulation of Multicellular Organismal Process | 9 | 33 | 1273 | 19416 | 0,000851 | 4,16 | 0,0235 |
| Response to Endogenous Stimulus | 9 | 33 | 1296 | 19416 | 0,000957 | 4,09 | 0,0258 |
| Odontogenesis | 4 | 33 | 125 | 19416 | 0,00113 | 18,83 | 0,0298 |
| Extracellular Matrix Assembly | 3 | 33 | 32 | 19416 | 0,00127 | 55,16 | 0,0327 |
| Response to Muscle Activity | 3 | 33 | 34 | 19416 | 0,00143 | 51,91 | 0,0356 |
| Response to Amino Acid | 4 | 33 | 136 | 19416 | 0,00144 | 17,30 | 0,0356 |
| Embryo Development | 8 | 33 | 1074 | 19416 | 0,00155 | 4,38 | 0,0376 |
| Negative Regulation of Peptidase Activity | 3 | 33 | 39 | 19416 | 0,00188 | 45,26 | 0,0447 |
| Response to Acid Chemical | 4 | 33 | 151 | 19416 | 0,00194 | 15,59 | 0,0453 |
| Negative Regulation of Proteolysis | 4 | 33 | 156 | 19416 | 0,00213 | 15,09 | 0,0488 |
| Cellular Response to Transforming Growth Factor Beta Stimulus | 4 | 33 | 165 | 19416 | 0,00249 | 14,26 | 0,0556 |
| Regeneration | 4 | 33 | 166 | 19416 | 0,00254 | 14,18 | 0,0556 |
| Multicellular Organismal Process | 20 | 33 | 6547 | 19416 | 0,00256 | 1,80 | 0,0556 |
| Response to Hormone | 7 | 33 | 871 | 19416 | 0,00267 | 4,73 | 0,0569 |
| Response to Transforming Growth Factor Beta | 4 | 33 | 172 | 19416 | 0,00281 | 13,68 | 0,0588 |
| Regulation of Multicellular Organismal Development | 9 | 33 | 1538 | 19416 | 0,00286 | 3,44 | 0,0589 |
| Response to Lipid | 7 | 33 | 900 | 19416 | 0,00314 | 4,58 | 0,0635 |
| Embryo Implantation | 3 | 33 | 51 | 19416 | 0,00319 | 34,61 | 0,0635 |
| Regulation of Multicellular Organismal Process | 13 | 33 | 3194 | 19416 | 0,0034 | 2,39 | 0,0664 |
| Cartilage Development | 4 | 33 | 185 | 19416 | 0,00344 | 12,72 | 0,0664 |
| Response to Peptide | 7 | 33 | 932 | 19416 | 0,00373 | 4,42 | 0,0709 |
| Response to Stress | 14 | 33 | 3689 | 19416 | 0,0038 | 2,23 | 0,071 |
| Response to Oxygen-Containing Compound | 9 | 33 | 1677 | 19416 | 0,00488 | 3,16 | 0,0898 |
| Cellular Response to Chemical Stimulus | 10 | 33 | 2066 | 19416 | 0,00496 | 2,85 | 0,09 |
| Cellular Response to Endogenous Stimulus | 7 | 33 | 994 | 19416 | 0,00512 | 4,14 | 0,0914 |
| Negative Regulation of Cell-Substrate Adhesion | 3 | 33 | 68 | 19416 | 0,0056 | 25,96 | 0,0981 |
| Response to Chemical | 14 | 33 | 3856 | 19416 | 0,00565 | 2,14 | 0,0981 |
| Regulation of Peptidase Activity | 3 | 33 | 73 | 19416 | 0,00643 | 24,18 | 0,11 |
| Response to Mechanical Stimulus | 4 | 33 | 233 | 19416 | 0,00655 | 10,10 | 0,11 |
| Negative Regulation of Developmental Process | 7 | 33 | 1053 | 19416 | 0,00675 | 3,91 | 0,112 |
| Connective Tissue Development | 4 | 33 | 244 | 19416 | 0,00743 | 9,65 | 0,122 |
| Regulation of Developmental Process | 11 | 33 | 2642 | 19416 | 0,00798 | 2,45 | 0,129 |
| Regulation of Cell Population Proliferation | 9 | 33 | 1830 | 19416 | 0,00823 | 2,89 | 0,132 |
| Regulation of Cell Migration | 7 | 33 | 1103 | 19416 | 0,00842 | 3,73 | 0,133 |
| Negative Regulation of Hydrolase Activity | 3 | 33 | 89 | 19416 | 0,00942 | 19,83 | 0,147 |
| Cellular Response to Oxygen-Containing Compound | 7 | 33 | 1149 | 19416 | 0,0102 | 3,58 | 0,156 |
| Biological Regulation | 29 | 33 | 13191 | 19416 | 0,0103 | 1,29 | 0,156 |
| Regulation of Biological Quality | 12 | 33 | 3194 | 19416 | 0,0104 | 2,21 | 0,157 |
| Response to Nutrient Levels | 5 | 33 | 528 | 19416 | 0,0106 | 5,57 | 0,158 |
| Regulation of Cell Motility | 7 | 33 | 1169 | 19416 | 0,0111 | 3,52 | 0,162 |
| **Direct Ossification** | 2 | 33 | 7 | 19416 | 0,0115 | 168,10 | 0,164 |
| **Intramembranous Ossification** | 2 | 33 | 7 | 19416 | 0,0115 | 168,10 | 0,164 |
| Skin Development | 4 | 33 | 291 | 19416 | 0,012 | 8,09 | 0,169 |
| Negative Regulation of Plasminogen Activation | 2 | 33 | 8 | 19416 | 0,0131 | 147,09 | 0,181 |
| Regulation of Locomotion | 7 | 33 | 1213 | 19416 | 0,0131 | 3,40 | 0,181 |
| Defense Response | 8 | 33 | 1589 | 19416 | 0,0133 | 2,96 | 0,181 |
| Response to Retinoic Acid | 3 | 33 | 109 | 19416 | 0,0139 | 16,19 | 0,184 |
| Biomineral Tissue Development | 3 | 33 | 109 | 19416 | 0,0139 | 16,19 | 0,184 |
| **Ossification** | 4 | 33 | 308 | 19416 | 0,014 | 7,64 | 0,184 |
| **Bone Trabecula Formation** | 2 | 33 | 9 | 19416 | 0,0147 | 130,75 | 0,193 |
| Response to Cytokine | 6 | 33 | 916 | 19416 | 0,0161 | 3,85 | 0,209 |
| Heart Development | 5 | 33 | 600 | 19416 | 0,0164 | 4,90 | 0,209 |
| Regulation of Biological Process | 28 | 33 | 12777 | 19416 | 0,0169 | 1,29 | 0,214 |
| Response to Estradiol | 3 | 33 | 123 | 19416 | 0,0174 | 14,35 | 0,218 |
| Positive Regulation of Cell Differentiation | 6 | 33 | 940 | 19416 | 0,0179 | 3,76 | 0,221 |
| Positive Regulation of Cell-Substrate Adhesion | 3 | 33 | 130 | 19416 | 0,0194 | 13,58 | 0,234 |
| Bone Trabecula Morphogenesis | 2 | 33 | 12 | 19416 | 0,0196 | 98,06 | 0,234 |
| Luteinization | 2 | 33 | 12 | 19416 | 0,0196 | 98,06 | 0,234 |
| Skin Morphogenesis | 2 | 33 | 12 | 19416 | 0,0196 | 98,06 | 0,234 |
| Enzyme-Linked Receptor Protein Signaling Pathway | 5 | 33 | 649 | 19416 | 0,0212 | 4,53 | 0,25 |
| System Process | 9 | 33 | 2176 | 19416 | 0,022 | 2,43 | 0,257 |
| Ameboidal-Type Cell Migration | 3 | 33 | 142 | 19416 | 0,0228 | 12,43 | 0,264 |
| Positive Regulation of Multicellular Organismal Process | 8 | 33 | 1773 | 19416 | 0,023 | 2,65 | 0,264 |
| Protein Hydroxylation | 2 | 33 | 16 | 19416 | 0,0261 | 73,55 | 0,296 |
| Regulation of Cellular Process | 27 | 33 | 12386 | 19416 | 0,0265 | 1,28 | 0,298 |
| Response to Uv | 3 | 33 | 158 | 19416 | 0,0278 | 11,17 | 0,309 |
| Positive Regulation of Developmental Process | 7 | 33 | 1437 | 19416 | 0,0279 | 2,87 | 0,309 |
| Response to Stimulus | 21 | 33 | 8582 | 19416 | 0,0285 | 1,44 | 0,311 |
| Negative Regulation of Cellular Process | 16 | 33 | 5739 | 19416 | 0,0286 | 1,64 | 0,311 |
| Regulation of Plasminogen Activation | 2 | 33 | 18 | 19416 | 0,0293 | 65,37 | 0,311 |
| Negative Regulation of Angiogenesis | 3 | 33 | 163 | 19416 | 0,0295 | 10,83 | 0,311 |
| Regulation of Apoptotic Signaling Pathway | 4 | 33 | 411 | 19416 | 0,0297 | 5,73 | 0,311 |
| Regulation of Protein Metabolic Process | 8 | 33 | 1868 | 19416 | 0,0297 | 2,52 | 0,311 |
| Negative Regulation of Blood Vessel Morphogenesis | 3 | 33 | 165 | 19416 | 0,0301 | 10,70 | 0,313 |
| Negative Regulation of Vasculature Development | 3 | 33 | 167 | 19416 | 0,0308 | 10,57 | 0,317 |
| Regulation of Cell Growth | 4 | 33 | 419 | 19416 | 0,0312 | 5,62 | 0,318 |
| Regulation of Proteolysis | 4 | 33 | 426 | 19416 | 0,0325 | 5,52 | 0,329 |
| Negative Regulation of Protein Metabolic Process | 5 | 33 | 747 | 19416 | 0,0333 | 3,94 | 0,334 |
| Response to Hyperoxia | 2 | 33 | 22 | 19416 | 0,0357 | 53,49 | 0,352 |
| Response to Peptide Hormone | 4 | 33 | 442 | 19416 | 0,0357 | 5,32 | 0,352 |
| Response to Reactive Oxygen Species | 3 | 33 | 182 | 19416 | 0,0361 | 9,70 | 0,353 |
| Female Pregnancy | 3 | 33 | 183 | 19416 | 0,0364 | 9,65 | 0,354 |
| Blood Circulation | 4 | 33 | 448 | 19416 | 0,0369 | 5,25 | 0,356 |
| Negative Regulation of Protein Processing | 2 | 33 | 23 | 19416 | 0,0372 | 51,16 | 0,356 |
| Positive Regulation of Biological Process | 17 | 33 | 6479 | 19416 | 0,0379 | 1,54 | 0,36 |
| Cellular Response to Vitamin | 2 | 33 | 24 | 19416 | 0,0388 | 49,03 | 0,361 |
| Cell Differentiation | 12 | 33 | 3857 | 19416 | 0,0392 | 1,83 | 0,361 |
| Cellular Developmental Process | 12 | 33 | 3860 | 19416 | 0,0394 | 1,83 | 0,361 |
| Response to Xenobiotic Stimulus | 4 | 33 | 460 | 19416 | 0,0394 | 5,12 | 0,361 |
| Negative Regulation of Biological Process | 16 | 33 | 5957 | 19416 | 0,0395 | 1,58 | 0,361 |

Note: DEPs: Differential expressed proteins. Hvs-CM: Healthy volunteer-conditioned media.
